# Effect of Acupuncture on Cognitive Function in Patients With Post‐Stroke Cognitive Impairment: A Systematic Review and Meta‐Analysis

**DOI:** 10.1002/brb3.70075

**Published:** 2024-10-14

**Authors:** Ziyan Luo, Wenxuan Li, Jieting Jiang, Jie Sun, Mingyue Zhang, Yaqing Zhang, Lu Dong, Kunpeng Li, Caiqin Wu

**Affiliations:** ^1^ School of Nursing Shanghai University of Traditional Chinese Medicine Shanghai China; ^2^ School of Acupuncture‐Moxibustion and Tuina Shanghai University of Traditional Chinese Medicine Shanghai China; ^3^ School of Nursing Shanghai Jiao Tong University Shanghai China; ^4^ School of Exercise and Health Shanghai University of Sport Shanghai China

**Keywords:** acupuncture, cognitive function, meta‐analysis, post‐stroke cognitive impairment, systemic review

## Abstract

**Aims and objective:**

To investigate the impact of acupuncture on post‐stroke cognitive impairment (PSCI).

**Background:**

PSCI is a major barrier to stroke patients’ rehabilitation, and acupuncture is one of the treatments. However, the benefit of acupuncture on PSCI is unclear.

**Design:**

A meta‐analysis and systematic review of randomized controlled trials (RCTs).

**Methods:**

Up to February 1, 2024, databases in PubMed, MEDLINE, Scopus, Embase, Web of Science, Cochrane Library, China National Knowledge Infrastructure, VIP, and Wanfang Data were searched. The risk of bias was investigated using the *Cochrane Handbook* for systematic reviews of treatments. Random‐effect and fix‐effect models were used to report the effects.

**Results:**

There were 29 randomized clinical trials with 2477 participants included. The findings demonstrated that the Mini‐Mental State Examination (MMSE) and the Montreal Cognitive Assessment (MoCA) scores were higher in the acupuncture group than medicine group (mean difference [MD] = 1.74, 95% confidence interval (CI) CI [1.26, 2.23], *I* ^2^ = 59%, *p* *<* 0.01). Compared to medicine group, the Loewenstein Occupational Therapy Cognitive Assessment (LOTCA) score exhibited a significant decrease and demonstrated improvement in the acupuncture group. Statistically significant outcomes were observed in the Barthel Index scores and P300 event‐related potential (ERP). According to subgroup analysis, acupuncture was superior to conventional therapy for improving cognitive function in PSCI patients at 4 weeks after treatment.

**Conclusion:**

Acupuncture therapy has shown promise in ameliorating cognitive deficits and enhancing daily functional abilities in individuals diagnosed with PSCI. But future research should focus on the duration and implement large sample, high‐quality RCTs.

**Relevance to clinical practice:**

Clinical workers in practical clinical work can select appropriate acupoints according to the actual conditions of patients, as well as confirm the treatment course of PSCI patients, while paying attention to observing and evaluating the therapeutic efficacy of acupuncture, to improve the health outcomes of patients in a patient‐centered way.

## Graphical abstract

This study systematically reviewed and meta‐analyzed randomized controlled trials to evaluate the impact of acupuncture on post‐stroke cognitive impairment (PSCI). The analysis included 29 trials with 2477 participants, indicating that acupuncture could significantly improve cognitive function as measured by Mini‐Mental State Examination and Montreal Cognitive Assessment scores, and enhance daily living abilities as shown by Barthel Index scores and P300 event‐related potential. Subgroup analysis suggested that acupuncture was particularly effective at 4 weeks post‐treatment. While promising, the study calls for future research with larger samples and longer treatment durations to solidify these findings.

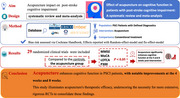

## 1 Introduction

Post‐stroke cognitive impairment (PSCI) is a pervasive neurological condition that emerges beyond the 6‐month mark following a stroke event (Huang et al. 2022). It is characterized by a significant deterioration in cognitive functions, which profoundly affects the quality of life and survival times of over 70% of stroke survivors (Balas and McNett 2022; Rost et al. 2022; Sun, Tan, and Yu 2014). The alarming prevalence and disability rate associated with PSCI, coupled with the increasing incidence of stroke, particularly in the context of an aging population in China, underscore the urgency of addressing this public health challenge (Tu et al. 2023). The latest data indicate a 2.6% prevalence among adults aged 40 and above, with an annual incidence rate of 505.2 per 100,000 person‐years and a mortality rate of 343.4 per 100,000 person‐years (Jing, Bao, and Seet 2023; Wang et al. 2022). The cognitive function assessment of PSCI patients is an essential component of clinical management, typically conducted using the Montreal Cognitive Assessment (MoCA) and the Mini‐Mental State Examination (MMSE) (Quinn et al. 2021). According to guidelines established by the European Stroke Organization and the European Academy of Neurology, these assessment tools are recommended for evaluating the cognitive function of PSCI (Quinn et al. 2021). These instruments provide vital information to healthcare professionals, aiding them in understanding the cognitive status of patients and formulating personalized treatment plans. Moreover, the guidelines emphasize the importance of considering the specific circumstances of patients, including the results of cognitive function assessments, to achieve optimal therapeutic outcomes.

Current therapeutic approaches for PSCI encompass both pharmacological and non‐pharmacological interventions (Lingo et al. 2020; Teng et al. 2017). Although medications such as cholinesterase inhibitors and memantine have been considered, their efficacy in forestalling cognitive decline post‐stroke remains contentious, with evidence of limited effectiveness and potential for adverse effects over time. In contrast, non‐pharmacological interventions, notably acupuncture, have been increasingly recognized for their potential benefits, as highlighted in European guidelines advocating for their broader application (Birch and Robinson 2022; Wang et al. 2022; Lin et al. 2022). The exploration of acupuncture as a treatment modality is particularly timely, given the dearth of satisfactory pharmacological options and the growing interest in holistic approaches to neurological rehabilitation.

In previous studies, there have been diverse original research findings on the efficacy of acupuncture for PSCI, leading to some controversy regarding its effectiveness. Current research also includes meta‐analysis examining the effects of acupuncture on PSCI, such as comparisons of different acupuncture methods. The role of acupuncture in PSCI has been examined in several randomized controlled trials (RCTs), yielding a spectrum of outcomes. Some studies suggest that acupuncture, as part of a multimodal intervention, may enhance cognitive and motor functions in stroke survivors (Zhan et al. 2017). However, the heterogeneity in study design, sample size, and acupuncture protocols has led to inconsistent findings and a pressing need for further investigation. A synthesis of existing RCTs reveals a dichotomy in the efficacy of acupuncture, with some studies reporting positive outcomes while others show no significant benefits (Chen et al. 2020; Zhan et al. 2017). This variance may be attributed to differences in treatment protocols, including the specific acupoints used, the duration and frequency of sessions, and the integration with other therapeutic modalities.

Given the discordant results and the evolving landscape of acupuncture research, there is a compelling case for an updated systematic review and meta‐analysis. Such an analysis would not only re‐evaluate the clinical efficacy and safety of acupuncture in PSCI but also explore the impact of varying treatment durations and protocols on cognitive outcomes. However, our study builds upon this foundation by specifically comparing the effects of different intervention durations on cognitive function in PSCI. By conducting a meta‐analysis, this study aims to provide a more nuanced understanding of acupuncture's role in PSCI management, contributing to the evidence base that informs clinical practice and future research directions.

## 2 Methods

The review was conducted and reported according to the PRISMA (Preferred Reporting Items for Systematic Reviews and Meta‐analysis) guidelines, as shown in Table S1. A complete descriptive report of this study was submitted to INPLASY prior to formal commencement (Registration number: INPLASY2022110115).

### 2.1 Search Strategy

Several databases were screened, including PubMed, MEDLINE, Scopus, Embase, Web of Science, the Cochrane Library, China National Knowledge Infrastructure (CNKI), VIP, and Wanfang Data, from database inception to February 1, 2024. The period covered ranged from the creation of the index to the date of query, and references were identified retrospectively. The search terms included (cognition OR cognitive dysfunction OR cognitive disorders OR cognitive impairments OR cerebrovascular accident OR cerebrovascular disorders OR cerebrovascular disorders OR cerebrovascular disease OR stroke) AND (acupuncture OR acupuncture therapy OR acupuncture point OR acupuncture analgesia) AND RCTs. The search strategy was peer‐reviewed by a second information specialist. The full search strategy is included in the Supporting Information section. The full text of relevant articles was retrieved. Inclusion criteria were assessed on a case‐by‐case basis, and disagreements were resolved by mutual agreement. Furthermore, the literature references of the included articles as well as additional previously published meta‐analyses were examined. Totally two separate assessors (Z.Y. LUO and W.X. LI) extensively scanned and extracted the included trials. Predefined query patterns from PubMed, as an example, were included in the protocol (Ye et al. 2017). Various databases were consulted with the relevant keywords. To ensure the quality of the studies analyzed, to reduce publication bias, and to ensure the comprehensiveness of the articles retrieved, a manual search of the gray literature was chosen for this review (Page et al. 2021).

### 2.2 Inclusion and Exclusion Criteria

Studies were eligible if they met all of the following criteria:

The selection of eligibility criteria was guided by the Population, Intervention, Comparison, and Outcome (PICO) framework.

Study design: RCT.

Population: Patients with PSCI with clear and standardized diagnostic criteria.

Interventions: One group was treated with acupuncture (e.g., basic acupuncture, electroacupuncture, and others), compared with the control group.

Comparisons: Received other non‐acupuncture treatments. Apart from acupuncture, the other therapies were supposed to be the right ones for both groups, and there were no restrictions on the course of treatment.

Outcome: Post‐treatment cognitive function, including MMSE, MoCA, Loewenstein Occupational Therapy Cognitive Assessment (LOTCA), P300 event‐related potential (ERP), and quality of life (e.g., Barthel Index, BI).

Exclusion criteria: Research protocols, ongoing studies, reviews, animal experiments, full text was not available, and conference abstracts were excluded.

### 2.3 Study Selection

Totally two researchers independently screened and reviewed according to PRISMA standards (Page et al. 2021). Initially, the researchers embarked upon a meticulous screening process, scrutinizing the titles and abstracts of potential articles to exclude those irrelevant to the theme. Subsequently, the entire content of selected documents underwent comprehensive evaluation. Only those that satisfied the criteria for the trials were included in the analysis. If there was any discrepancy between the two investigators, a rigorous discussion ensued until a consensus was reached, with the involvement of a third investigator as necessary.

### 2.4 Data Extraction

A thorough and independent peer review of these publications was carried out by two peer reviewers. In the event of a difference of opinion, a neutral third examiner mediated. Information extracted included: title, first author's details, main features of included trials (population, patient age, interventions, etc.), publication date, sources of bias (methods of allocation, blindness, etc.), and outcomes (MMSE, MoCA, and overall efficacy). Where the details listed above were ambiguous, the author of the original text was contacted.

### 2.5 Risk of Bias

The Cochrane Risk of Bias Tool 2.0 (RoB2) was used to assess the risk of bias in the included trials. This comprehensive tool assesses potential bias in the following areas: (1) the randomization process, (2) deviations from the intended interventions, (3) cases of missing outcome data, (4) the methodology of outcome measurement, and (5) the selection criteria for selection criteria for reported outcomes. Each domain was classified according to the risk of bias, with possible ratings of low, some concern, or high. This systematic approach ensures a rigorous and consistent assessment of the potential bias present in each study, thereby increasing the reliability and validity of the review. The risk of bias of each included study was independently assessed by two researchers (Z.Y. LUO and W.X. LI) to ensure the highest methodological standards in our systematic review and meta‐analysis. Any discrepancies or inconsistencies identified during this independent assessment were first discussed between the two researchers for resolution. If consensus could not be disagreement, a third (J.T. Jiang) impartial researcher was engaged to provide a definitive judgment. Additional information is provided in Table S2.

### 2.6 Data Synthesis

Heterogeneity was calculated as *I* ^2^. There was no significant heterogeneity across studies was found if *p* ≥ 0.1 in *χ* ^2^ test and *I* ^2^ ≤ 50%, and the fixed effect model was used (Kovács et al. 2022; Mantel and Haenszel 1959). Otherwise, the random effect model was applied to analyze the sources of heterogeneity through subgroups (Bryant et al. 2022; DerSimonian and Laird 1986). When the outcome measures in this study were for continuous items, the mean difference (MD) was reported here as the magnitude of the effect. The odds ratio (OR) and associated 95% confidence interval (CI) were used as the effect size when the variable was binary. Forest and funnel plots were generated using Review Manager 5.4 (Higgins et al. 2019), with *I* ^2^ used to assess the degree of heterogeneity and the *p* value or 95% CI used to assess publication bias. The overall standard and strength of the effects of interventions were assessed using the *Cochrane Handbook for Systematic Reviews of Interventions* (Higgins 2011). Where data can be combined, a meta‐analysis is used, and where data cannot be combined, a descriptive approach is used for a systematic review. Lastly, to explore the sources of heterogeneity and gain a better insight into the effects of the interventions, subgroup analysis and meta‐regression models were adjusted to explore the sensitivity of the effects of the interventions. Funnel plots and Egger bias tests were used to assess publication bias.

## 3 Results

### 3.1 Study Selection

**FIGURE 1:**
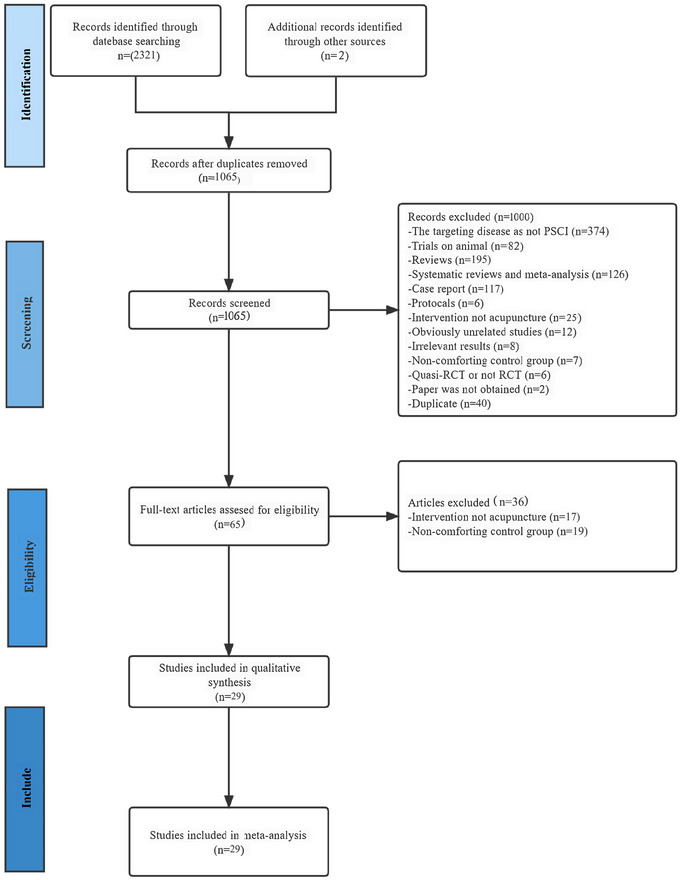
Preferred reporting items for systematic reviews and meta‐analyses (PRISMA) flow diagram of the literature inclusion process. PSCI, post‐stroke cognitive impairment; RCT, randomized controlled trial.

In total, 2321 articles were originally eligible. Following a step‐by‐step screening process, ultimately 29 studies were considered eligible; the flow diagram of the article testing process and output is illustrated in Figure 1. In a number of qualifying studies, 10 of the studies (Chen et al. 2020; Chen et al. 2016; Ding and Zhou 2020; Huang et al. 2021; Jiang et al. 2016; Liu et al. 2016; Wang et al. 2016; Xiong et al. 2020; Yang et al. 2015; Yao and Gong 2020) were English versions, and 19 of them (Cao 2015; Zheng et al. 2021; Chen, Lu, and Zhou 2018; Feng 2013; Jiang 2011; Kang 2011; Li, Ban, and Chen 2023; Lin et al. 2014; Liu 2015; Su et al. 2023; Sun 2013; Wang and Li 2015; Wei, Liu, and Zeng 2023; Yang et al. 2017; Yang 2011; Yang and Zhang 2019; Yuan et al. 2022; Yuan et al. 2022; Zeng et al. 2015; Zhang et al. 2022; Zhang 2014) were Chinese versions, of which acupuncture was used in the experimental group whereas medication was used in the control group.

### 3.2 Study Characteristics

A total of 29 studies were eligible, with 2477 participants registered and 976 participants (39.4%) women. Totally 13 studies (Ding and Zhou 2020; Huang et al. 2021; Jiang et al. 2016; Jiang 2011; Kang 2011; Wei, Liu, and Zeng 2023; Yang 2011; Yao and Gong 2020; Yuan et al. 2022; Zeng et al. 2015) examined hemorrhagic stroke and ischemic stroke as the study population; otherwise, 3 studies (Chen et al. 2016; Chen, Lu, and Zhou 2018; Wang, Yang, and Zhang 2016) included participants with ischemic stroke, and only 1 study (Liu 2015) included participants with stroke during convalescence. In 17 studies (Chen et al. 2016; Chen, Lu, and Zhou 2018; Ding and Zhou 2020; Feng 2013; Jiang et al. 2016; Jiang 2011; Kang 2011; Lin et al. 2014; Sun 2013; Wang and Li 2015; Wei, Liu, and Zeng 2023; Xiong et al. 2020; Yang et al. 2017; Yang et al. 2015; Yang 2011; Yao and Gong 2020; Zeng et al. 2015), conventional western care was used as the control group, and in 6 studies (Cao 2015; Chen et al. 2020; Liu 2015; Liu et al. 2016; Wang et al. 2016; Zhang 2014), medication was used as the treatment, with 3 studies (Liu 2015; Wang et al. 2016; Zhang 2014) using nimodipine, 1 study (Cao 2015) using oxiracetam, and 1 study (Liu et al. 2016) using donepezil hydrochloride. Furthermore, four studies used sham acupuncture as a control group (Zheng et al. 2021; Huang et al. 2021; Li, Ban, and Chen 2023; Su et al. 2023). In aggregate, the trial group lost 40 participants, and the control group lost 33 participants. Among them, only one study investigated the impact of acupuncture on depression levels in patients with PSCI, yielding non‐statistically significant results (*p* = 0.89) (Huang et al. 2021). The PRISMA flowchart of the literature inclusion process is displayed in Table 1.

**TABLE 1:** Basic characteristics of eligible randomized controlled trials (RCTs).

Eligible articles	Sample size	Gender Male:Female	Age ($\bar{x}$ ± s) (years)		The type of stroke	Course (days)	Intervention		Treatment courses and frequency	Outcomes	Dropout or withdrawal cases
	T/C	T/C	T	C			T	C			
Chen et al. (2020)	28/28	16:12/17:11	65.30 ± 6.80	64.50 ± 7.20	Not reported	Not reported	Cluster needling scalp acupuncture	Medication	4 weeks, 2/day	MoCA	Not reported
Jiang et al. (2016)	52/49	25:27/24:25	61.58 ± 9.71	60.53 ± 9.19	1. Hemorrhagic stroke 2. Ischemic stroke	T: 41.12 ± 21.71 C: 42.76 ± 16.00	Acupuncture at GV20, GV24	Routine western treatment	12 weeks, 5/week	1. MMSE 2.MoCA	T: 8 cases C: 2 cases
Chen et al. (2016)	125/125	74:51/74:51	62.52 ± 10.60	64.06 ± 10.54	Ischemic stroke	2–7	Electroacupuncture at LI15, LI11, ST36, SP6	Routine western treatment	3 weeks, 6/week	1. MMSE 2.MoCA	T: 5 cases C: 4 cases
Wang et al. (2016)	40/39	30:10/26:13	64.40 ± 7.70	60.60 ± 6.070	Ischemic stroke	15–180	Acupuncture at DU20, EX‐HN1, ST2, GB20, GB12, BL10, DU26, HT7, PC6, ST40, SP6, and LR3	Nimodipine alone	3 months, 2/week	MoCA	T: 2 cases C: 3 cases
Xiong et al. (2020)	35/35	20:15/17:18	63.00 ± 7.23	65.30 ± 8.52	Not reported	T: 2.13 ± 0.85 C: 2.51 ± 0.46	Acupuncture at GV 20, EX‐HN1, GB20, GV 24	Routine western treatment	8 weeks, 6/week	1. MMSE 2. LOTCA	T: 1 case C: 1 case
Yang et al. (2017)	30/30	17:13/16:14	66.9 0± 7.60	68.8 ± 8.10	Not reported	T: 15.50 ± 6.40 C: 13.9 ± 6.1	Long‐time needle retaining at DU20	Routine western treatment	4 weeks	MMSE	Not reported
Yao and Gong (2020)	30/30	19:11/21:90	54.60 ± 11.80	57.40 ± 12.80	1. Hemorrhagic stroke 2. Ischemic stroke	T: 2.50 ± 0.80 C: 2.40 ± 1.00	Acupoint combination of Jin's three‐needle. Main acupoints: BL23, SP6, CV6, BL17, ST40, CV12, BL40	Routine western treatment	4 weeks, 6/week	1. MMSE 2.MoCA	Not reported
Huan et al. (2021)	60/60	33:27/29:31	65.10 ± 7.50	64.6 ± 8.40	1. Hemorrhagic stroke 2. Ischemic stroke	Not reported	Electroacupuncture at GV20, GV29, GV24, GV26, GV17, EX‐HN1, GB20, HT7, SP6	Sham acupuncture	8 weeks	1. MoCA 2. MMSE 3. SDS 4. BI	Not reported
Sun (2013)	36/35	19:16/21:15	64.78 ± 6.32	64.01 ± 5.92	Not reported	T: 21.34 ± 25.33 C: 20.42 ± 25.19	Acupuncture at GV20, EX‐HN1, DU26, GB20, PC6, ST36, ST40, SP6	Routine western treatment	45 days, 1/day	1. MMSE 2. BI 3. SF‐36 4. NFDS	T: 3 cases C: 4 cases
Feng (2013)	40/40	31:9/31:9	52.13 ± 12.77	51.65 ± 12.47	Not reported	Not reported	Electroacupuncture at GV24, GV20	Routine western treatment	2 weeks, 1/day	1. MoCA 2. LOTCA 3. BI	T: 2 cases C: 2 cases
Liu (2015)	30/30	21:9/20:10	65.77 ± 8.24	67.00 ± 8.25	Apoplexy at convalescence stage	T: 3.85 ± 1.36 C: 3.70 ± 1.35	Electroacupuncture at DU19, GV20, DU21, DU24, GB13, DU26, PC6	Nimodipine alone	4 weeks, 5/week	1. MMSE 2. MoCA	Not reported
Kang (2011)	24/24	17:7/15:9	60.67 ± 6.93	62.71 ± 5.34	1. Hemorrhagic stroke 2. Ischemic stroke	T: 19.79 ± 6.78 C: 20.54 ± 5.40	Electroacupuncture at GV20, EX‐HN1	Routine western treatment	8 weeks, 1/day	1. MMSE 2. P300	Not reported
Zhang (2014)	30/30	16:14/17:13	71.27 ± 5.91	70.90 ± 5.62	Not reported	90–360	Acupuncture at GV20, EX‐HN1, DU24, GB20, SP6, CV4, KI03, HT7, PC6	Nimodipine alone	4 weeks, 5/week	1. MMSE 2. MoCA	Not reported
Cao (2015)	30/30	15:15/14:15	65.07 ± 7.06	64.07 ± 6.58	Not reported	T: 3.68 ± 1.71 C: 3.71 ± 1.48	Electroacupuncture at GV20, DU24, EX‐HN1, HT7, PC6	Oxiracetam	8 weeks, 6/week	1. MMSE 2. MoCA 3. BI	Not reported
Yang (2011)	20/20	11:9/10:10	59.00 ± 8.46	59.30 ± 8.42	1. Hemorrhagic stroke 2. Ischemic stroke	T: 65.80 ± 35.66 C: 66.0 ± 36.24	Electroacupuncture at GV20, GB20	Routine western treatment	8 weeks, 7/week	1. MMSE 2. P300	T: 0 case C: 1 case
Jiang (2011)	20/20	12:8/10:10	62.85 ± 5.67	61.75 ± 6.35	1. Hemorrhagic stroke 2. Ischemic stroke	T: 93.61 ± 41.84 C: 81.10 ± 41.15	Electroacupuncture at GV20, GV24	Routine western treatment	8 weeks, 7/week	1. MMSE 2. P300	Not reported
Zeng et al. (2015)	50/50	30:20/32:18	66.00 ± 12.00	68.00 ± 10.00	1. Hemorrhagic stroke 2. Ischemic stroke	T: 34.70 ± 2.90 C: 34.2 ± 2.9	Acupuncture at GV20, EX‐HN1, DU24, GV29, LI04, LR03	Routine western treatment	8 weeks, 5/week	1. MoCA 2. BI 3. FMA	Not reported
Lin et al. (2014)	30/29	18:12/19:10	65.00 ± 5.00	67.00 ± 7.00	Not reported	T: 29.85 ± 18.10 C: 30.05 ± 19.89	Acupuncture at GV20	Routine western treatment	28 days, 1/day	1. P300 2. MoCA	T: 0 case C: 1 case
Wang and Li (2015)	40/40	15:25/16:24	61.00 to75.00	59.00 to76.00	Not reported	T: 4–28 C: 5–29	Acupuncture at GV20, CV14, SJ05, SP10, ST36, GB34	Routine western treatment	4 weeks, 5/week	P300	Not reported
Ding and Zhou (2020)	44/44	24:20/25:19	60.55 ± 8.46	61.08 ± 7.59	1. Hemorrhagic stroke 2. Ischemic stroke	≥60	Xingnao Tongluo acupuncture	Routine western treatment	1 month, 1/day	1. BI 2. NCSE 3. HSP70 4. Cor	Not reported
Liu et al. (2016)	84/84	54:30/52:32	55.00 ± 7.00	56.00 ± 9.	Not reported	T: 2.9 ± 0.7 C: 3.20 ± 0.90	Acupuncture at DU24, BL10, DU26, PC6, HT7, GB20, GB12, DU20	Donepezil hydrochloride	56 days, 1/day	1. MMSE 2. P300	Not reported
Wei, Liu, and Zeng (2023)	40/40	25:15/27:14	53.18 ± 8.42	52.31 ± 7.87	1. Hemorrhagic stroke 2. Ischemic stroke	T: 2.14 ± 0.58 C: 2.26 ± 0.93	Acupuncture at DU20, EX‐HN1	Routine western treatment	4 weeks, 1/day	1. MMSE 2. MoCA 3. P300 4. BI	T: 0 case C: 0 case
Chen, Lu, and Zhou (2018)	60/60	45:15/42:18	65.43 ± 11.11	67.12 ± 10.98	1. Ischemic stroke	T: 35.61 ± 2.78 C: 35.11 ± 2.20	Acupuncture at DU20, EX‐HN1, GB20	Routine western treatment	8 weeks, 1/day	1. BI 2. FMA	T: 0 case C: 0 case
Yang et al. (2015)	36/36	25:11/21:15	66.00± 3.00	66 ± 3	Not reported	22 days to 4.2 months	Acupuncture at GV 20, EX‐HN 1, ST 2, GB 20, GB 12, BL 10, HT 7, PC 6, GV 26, SP 6, LR 3, ST 40	Routine western treatment	3 months, 3/day	1.MoCA	T: 0 case C: 0 case
Yang and Zhang (2019)	40/40	24:16/22:18	51.35 ± 7.30	51.72 ± 7.46	Not reported	T: 21.40 ± 5.38 C: 21.57 ± 5.54	Acupuncture at KI1, DU20	Routine western treatment	4 weeks, 2/day	1. MMSE 2. BI 3. FMA	T: 0 case C: 0 case
Yuan et al. (2022)	39/40	31:8/29:11	61.00 ± 8.00	59 ± 9	1. Hemorrhagic stroke 2. Ischemic stroke	T: 54.30 ± 37.70 C: 68.70 ± 50.00	Acupuncture at DU20, DU24	Routine western treatment	4 weeks, 1/day	1.MoCA 2.MMSE 3.BI	T: 1 case C: 0 case

Abbreviations: BI, Barthel Index; C, control group; Cor, cortisol; FMA, Fugl‐Meyer assessment; HSP70, serum heat shock protein; LOTCA, Loewenstein Occupational Therapy Cognitive Assessment; MMSE, Mini‐Mental State Examination; MoCA, Montreal Cognitive Assessment; NFDS, Neurological Function Deficit Scale; NIHSS, National Institute of Health Stroke Scale; P300, potential 300; SDS, Self‐Rating Depression Scale; SF‐36, short form‐36; T, treatment group.

### 3.3 Risk of Bias

**FIGURE 2:**
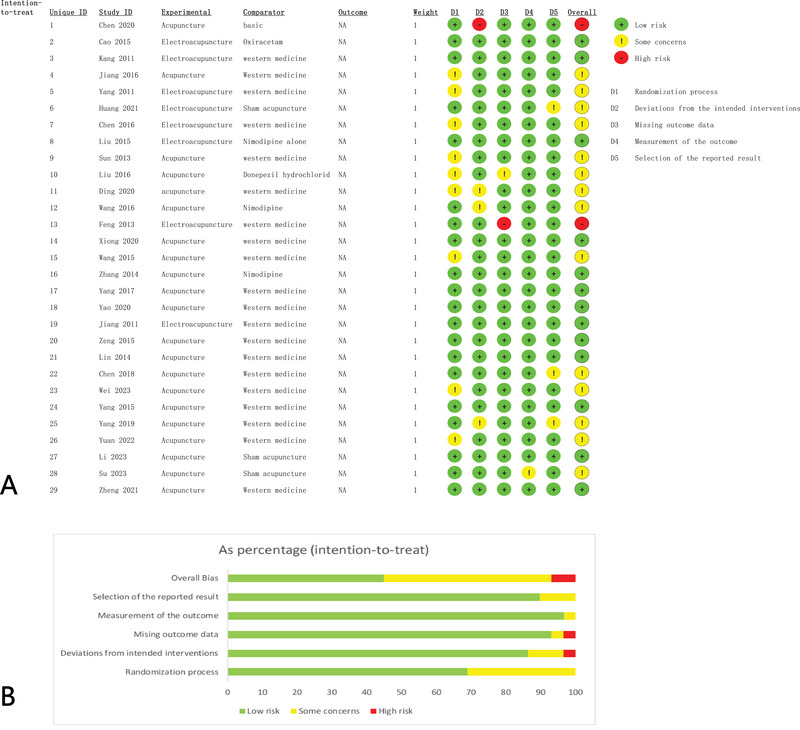
Risk of bias assessment of the included articles: (A) risk of bias graph; (B) risk of bias summary.

The risk of bias was estimated for all 29 studies, as illustrated in Figure 2. In 23 studies, the general level of the bias was considered to be unknown (Cao 2015; Chen et al. 2020; Chen et al. 2016; Ding and Zhou 2020; Feng 2013; Huang et al. 2021; Jiang, Yang, and Tao 2016; Jiang 2011; Kang 2011; Lin et al. 2014; Liu 2015; Liu et al. 2016; Sun 2013; Wang et al. 2016; Wei, Liu, and Zeng 2023; Xiong et al. 2020; Yang et al. 2017; Yang et al. 2015; Yang 2011; Yang and Zhang 2019; Yao and Gong 2020; Yuan et al. 2022; Zhang 2014). Potential sources of confounders include attrition bias, as three studies (Feng 2013; Sun 2013; Yang 2011) had partial outcome data and one study (Liu et al. 2016) had unidentified or missing values. In 15 studies (Cao 2015; Zheng et al. 2021; Chen et al. 2016; Feng 2013; Huang et al. 2021; Jiang 2011; Kang 2011; Li, Ban, and Chen 2023; Liu 2015; Liu et al. 2016; Su et al. 2023; Sun 2013; Wang et al. 2016; Xiong et al. 2020; Yang 2011), the order of randomization was properly determined and assignment was concealed. The results of the methodological quality assessment are listed in Table S3.

### 3.4 Effect on Cognition

#### 3.4.1 Effect on MoCA

**FIGURE 3:**
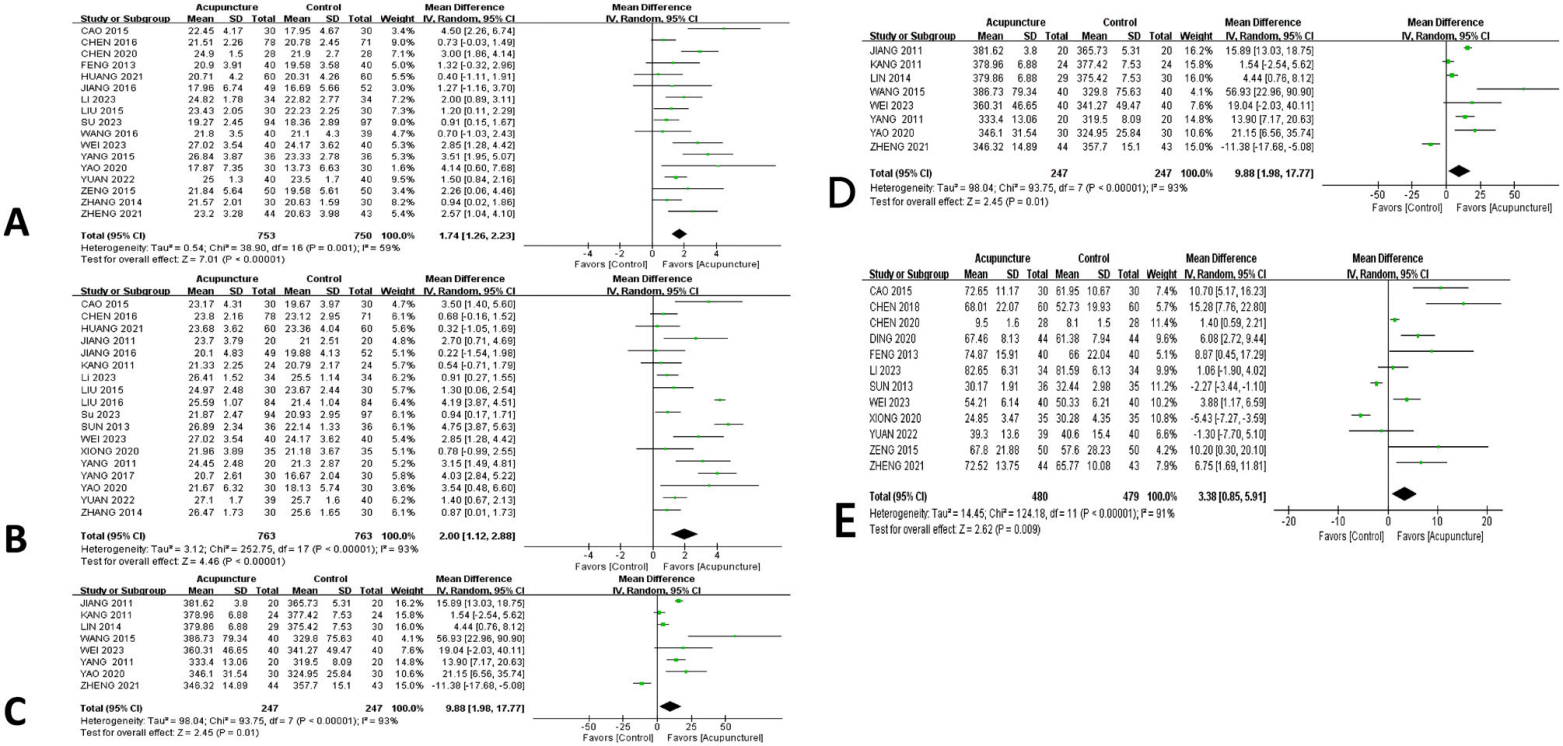
Forest plot of cognition in patients with PSCI: (A) forest plot of Montreal Cognitive Assessment (MoCA); (B) forest plot of Mini‐Mental State Examination (MMSE); (C) forest plot of P300 latency; (D) forest plot of P300 amplitude; (E) forest plot of Loewenstein Occupational Therapy Cognitive Assessment (LOTCA). CI, confidence interval; PSCI, post‐stroke cognitive impairment.

Altogether, 17 studies (Cao 2015; Zheng et al. 2021; Chen et al. 2020; Chen et al. 2016; Feng 2013; Huang et al. 2021; Jiang et al. 2016; Li, Ban, and Chen 2023; Liu 2015; Su, Lv, and Wu 2023; Wang et al. 2016; Wei, Liu, and Zeng 2023; Yang et al. 2015; Yao and Gong 2020; Yuan et al. 2022; Zeng et al. 2015; Zhang 2014) with 1503 cases were eligible for inclusion. The random effect model of meta‐analysis showed that acupuncture improved the MoCA score of the PSCI in comparison with routine western medical treatment, stating the MD (MD = 1.74, 95% CI [1.26, 2.23], *I* ^2^ = 59%, *p* < 0.01). The results are reported in Figure 3A.

#### 3.4.2 Effect on MMSE

Overall, 19 studies (Cao 2015; Chen et al. 2016; Huang et al. 2021; Jiang, Yang, and Tao 2016; Jiang 2011; Kang 2011; Li, Ban, and Chen 2023; Liu 2015; Liu et al. 2016; Su et al. 2023; Sun 2013; Wei, Liu, and Zeng 2023; Xiong et al. 2020; Yang et al. 2017; Yang 2011; Yang and Zhang 2019; Yao and Gong 2020; Yuan et al. 2022; Zhang 2014) with 1526 cases were selected for inclusion. When the random effects model of meta‐analysis was adopted, the results indicated that after acupuncture therapy, the MMSE score for PSCI patients had increased compared with the western medication, showing the MD (MD = 2.00, 95% CI [1.12,2.88], *I* ^2^ = 93%, *p* < 0.01); the result is presented in Figure 3B.

#### 3.4.3 Effect on P300 Latency and Amplitude

A total of 8 studies (Zheng et al. 2021; Jiang 2011; Kang 2011; Lin et al. 2014; Wang and Li 2015; Wei, Liu, and Zeng 2023; Yang 2011; Yao and Gong 2020) with 494 cases were reviewed. When the random effect model of meta‐analysis was applied to the P300 latency and the fixed effect model of meta‐analysis to the P300 amplitude, the data showed that acupuncture could improve the P300 latency score compared with routine western treatment, suggesting the MD (MD = 9.88, 95% CI [1.98,17.77] *I* ^2^ = 93%, *p* < 0.01), the result is depicted in Figure 3C. Meantime, the results showed that acupuncture could reduce the score of P300 amplitude comparatively with the western medication, indicating the MD (MD = 1.00, 95% CI [0.80,1.21], *I* ^2^ = 36%, *p* < 0.01); the finding is depicted in Figure 3D.

#### 3.4.4 Effect on LOTCA

In all, 2 studies (Feng 2013; Xiong et al. 2020) with 150 cases were involved. Applying the fixed effect model of meta‐analysis, the findings indicated that acupuncture therapy significantly reduced the LOTCA score in PSCI patients compared with western medication, indicating the MD (MD = 14.55, 95% CI [11.87,17.23], *I* ^2^ = 0%, *p* < 0.01); the result is as shown in Figure 3E.

### 3.5 Subgroup Analysis and Meta‐Regressions

The meta‐regression results showed that the sources contributing to high heterogeneity were identified through meta‐regression and ultimately found to be the time of intervention as a source of heterogeneity in the MMSE and therefore grouped by time of intervention in the subgroup analysis of the MMSE. The result of the meta‐regression is shown in Figure S1.

#### 3.5.1 Intervention Methods

**FIGURE 4:**
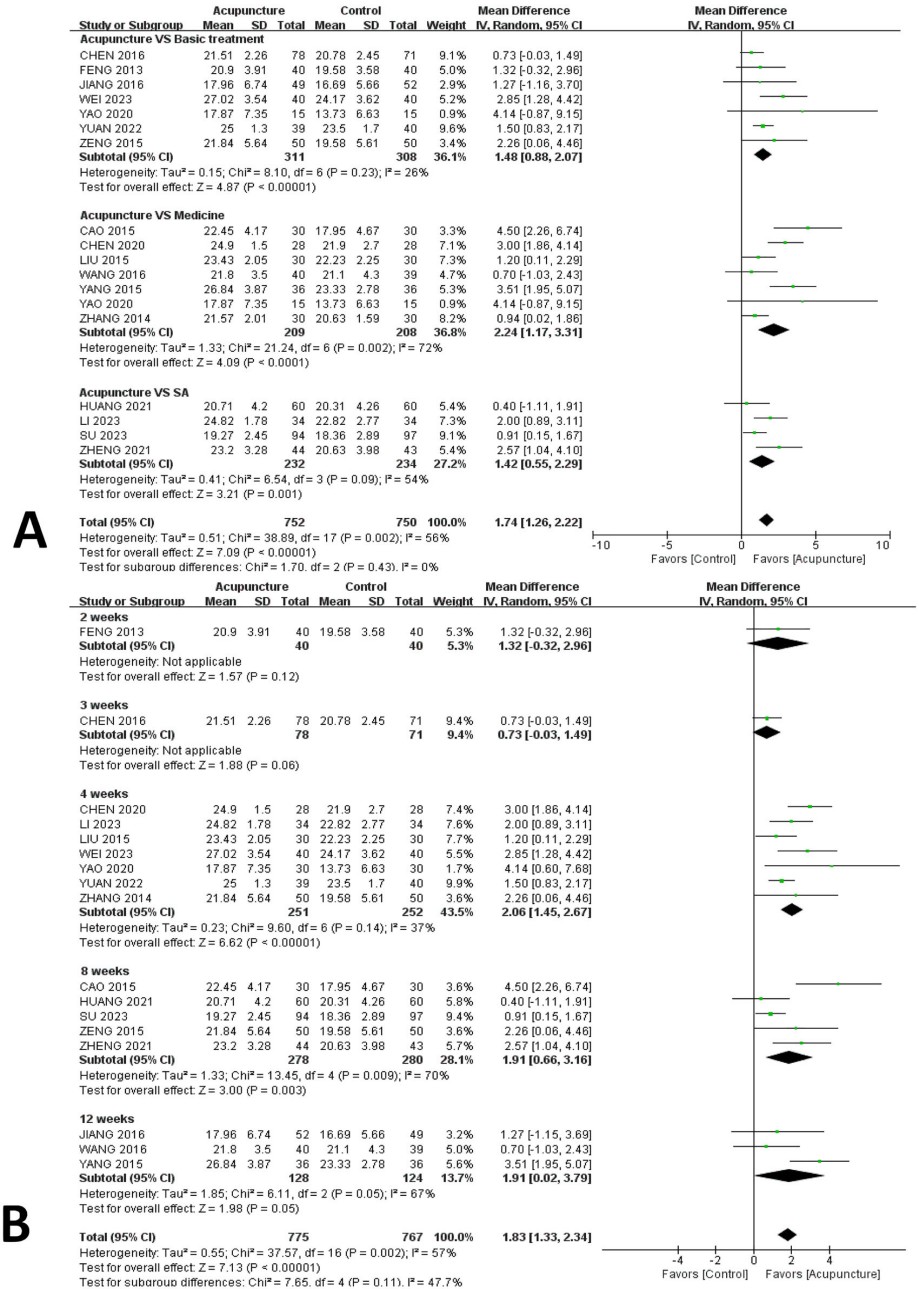
Subgroup analysis of changes in Montreal Cognitive Assessment. (A) subgroup analysis of different intervention methods; (B) subgroup analysis of different intervention times. CI, confidence interval.

Except for the basic treatment and sham group for the MoCA scores, all trials had an overall meaningful effect. The choice of medicines as controls was found to have a significant impact in 7 studies (Cao 2015; Chen et al. 2020; Liu 2015; Wang et al. 2016; Yang et al. 2015; Yao and Gong 2020; Zhang 2014) with 1036 cases and a random effect model (MD = 2.24, 95% CI [1.17,3.31], *I* ^2^ = 72%, *p* = 0.01). As the result of the subgroup analysis suggested that the reason for the differences in the articles was in the subgroup, this review used funnel plots to further explore the causes of the heterogeneity of the studies. A total of four studies (Zheng et al. 2021; Huang et al. 2021; Li, Ban, and Chen 2023; Su et al. 2023) chose sham acupuncture as a control in a random effect model. The findings have been presented in Figure 4A.

#### 3.5.2 Length of Intervention

As for the MoCA scores, this meta‐analysis demonstrated that, in people who were treated for 4, 8, and 12 weeks, there was acupuncture improved cognitive function (4 weeks: MD = 2.06, 95% CI, 1.45–2.67, *p* < 0.01; 8 weeks: MD = 1.91, 95% CI 0.66–3.16, *p* = 0.003; 12 weeks: MD = 1.91, 95% CI 0.02–3.79, *p* = 0.05). However, this was not significant at 2 and 3 weeks. The results are plotted in Figure 4B.

**FIGURE 5:**
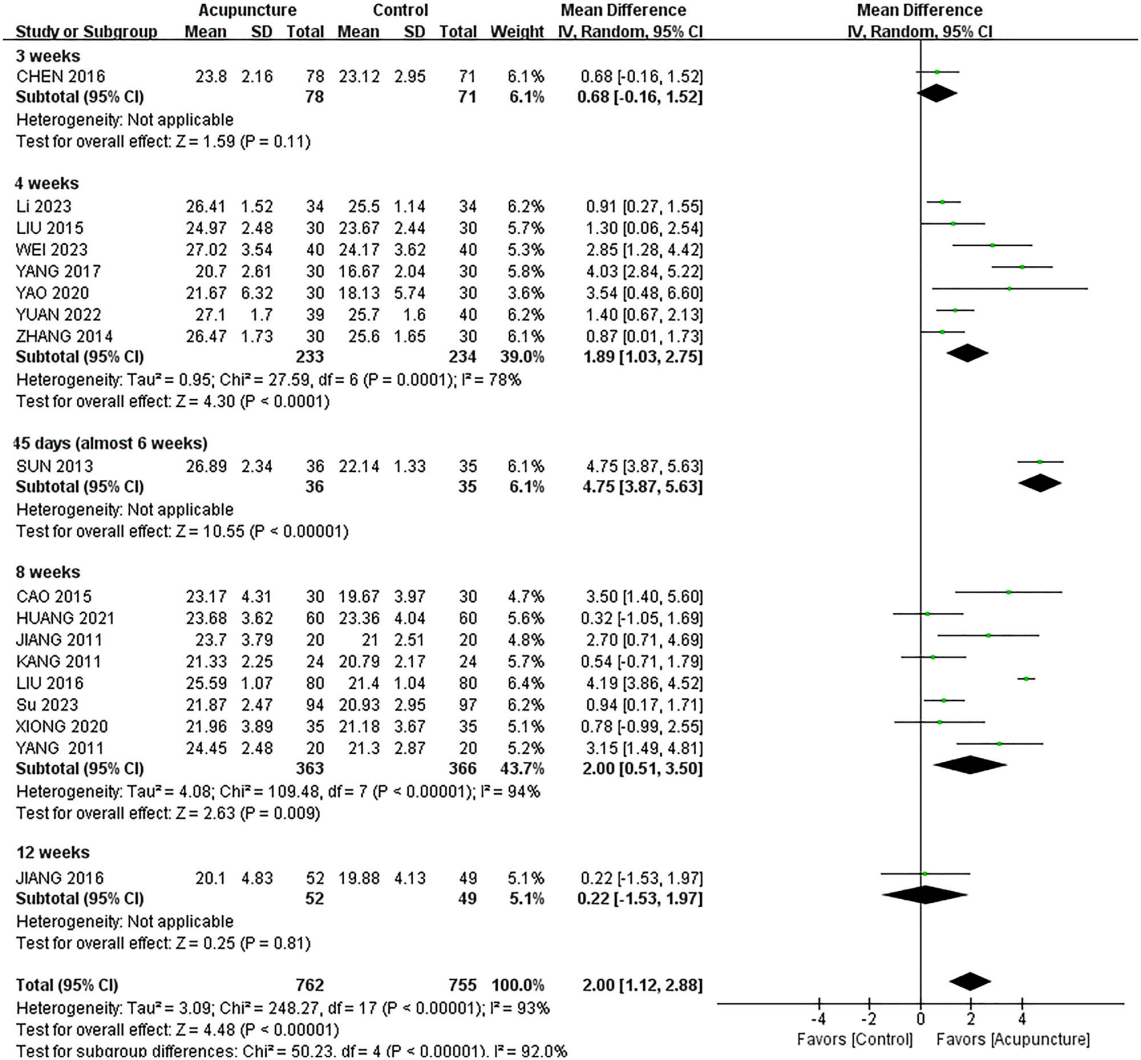
Subgroup analysis of different intervention times of changes in Mini‐Mental State Examination. CI, confidence interval.

Meanwhile, with regard to MMSE scores, it found that 4 and 8 weeks of intervention did have a marked impact (4 weeks: MD = 1.89; 95% CI, 1.03–2.75; *p* < 0.0001, *I* ^2^ = 78%; 8 weeks: MD = 2.00; 95% CI, 0.51–3.50; *p* = 0.009, *I* ^2^ = 94%). The results are presented in Figure 5.

### 3.6 Effect on Daily Living Ability

#### 3.6.1 Effect on BI

**FIGURE 6:**
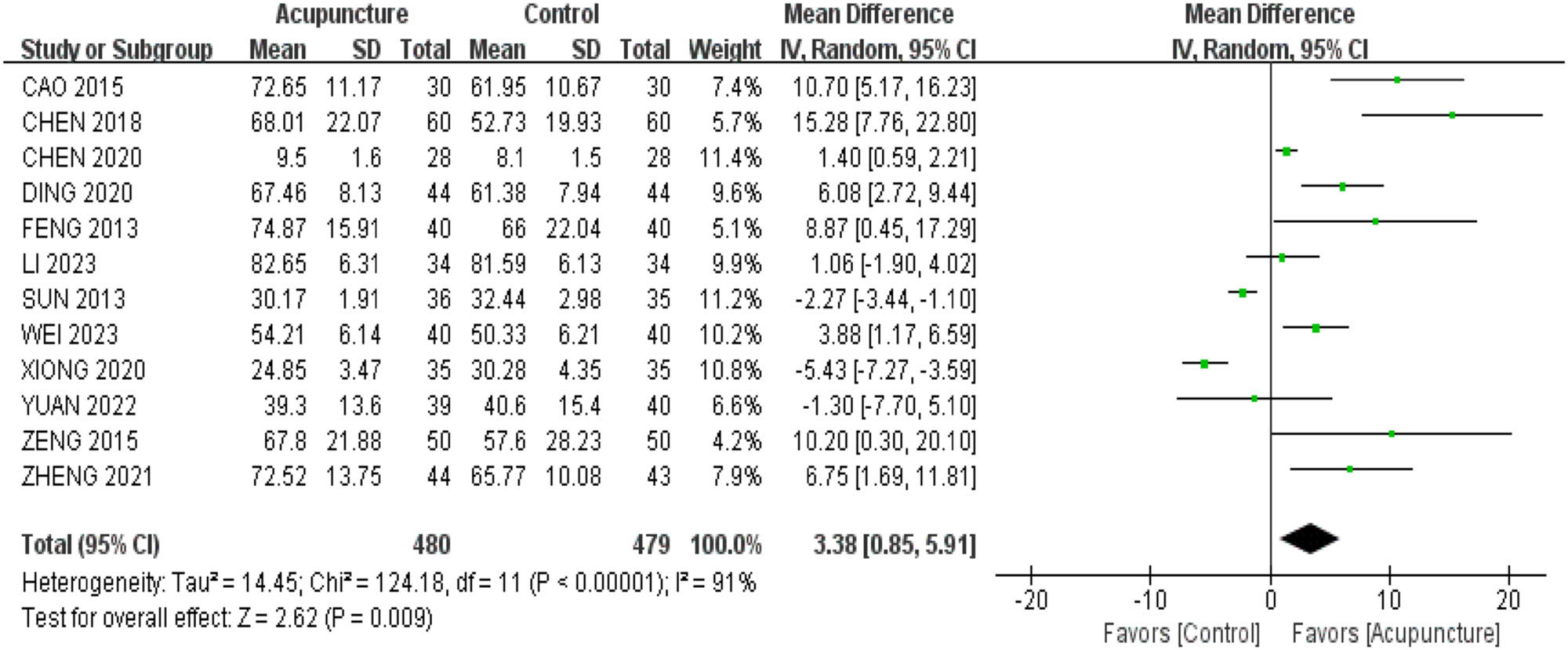
Forest plot of Barthel Index in patients with post‐stroke cognitive impairment.

Totally 11 studies (Cao 2015; Zheng et al. 2021; Chen et al. 2020; Chen, Lu, and Zhou 2018; Ding and Zhou 2020; Feng 2013; Sun 2013; Wei, Liu, and Zeng 2023; Xiong et al. 2020; Yang and Zhang 2019; Yuan et al. 2022; Zeng et al. 2015) with 959 cases were analyzed. According to the random effect model of meta‐analysis, the BI score for PSCI patients was significantly reduced compared with western medication, which indicated the MD (MD = 3.38, 95% CI [0.85,5.91], *I* ^2^ = 91%, *p* < 0.01); the result is presented in Figure 6A. The overview of the effects of acupuncture on cognition and motor function is presented in Table S4.

### 3.7 Effective Rate

A total of 10 studies (Cao 2015; Zheng et al. 2021; Chen, Lu, and Zhou 2018; Li, Ban, and Chen 2023; Liu 2015; Su et al. 2023; Sun 2013; Wei, Liu, and Zeng 2023; Yang et al. 2015; Yang and Zhang 2019) altogether provided efficacy figures for acupuncture treatment in 782 cases. When the fixed effect model of meta‐analysis was applied, the results showed the existence of statistical heterogeneity (*p* < 0.01, *I* ^2^ = 0%) in the efficacy rate of acupuncture therapy compared with conventional medicine and better healing benefits in the acupuncture group than the conventional medicine group. The results are plotted in Figure S2.

### 3.8 Publication Bias and Sensitive Estimate

**FIGURE 7:**
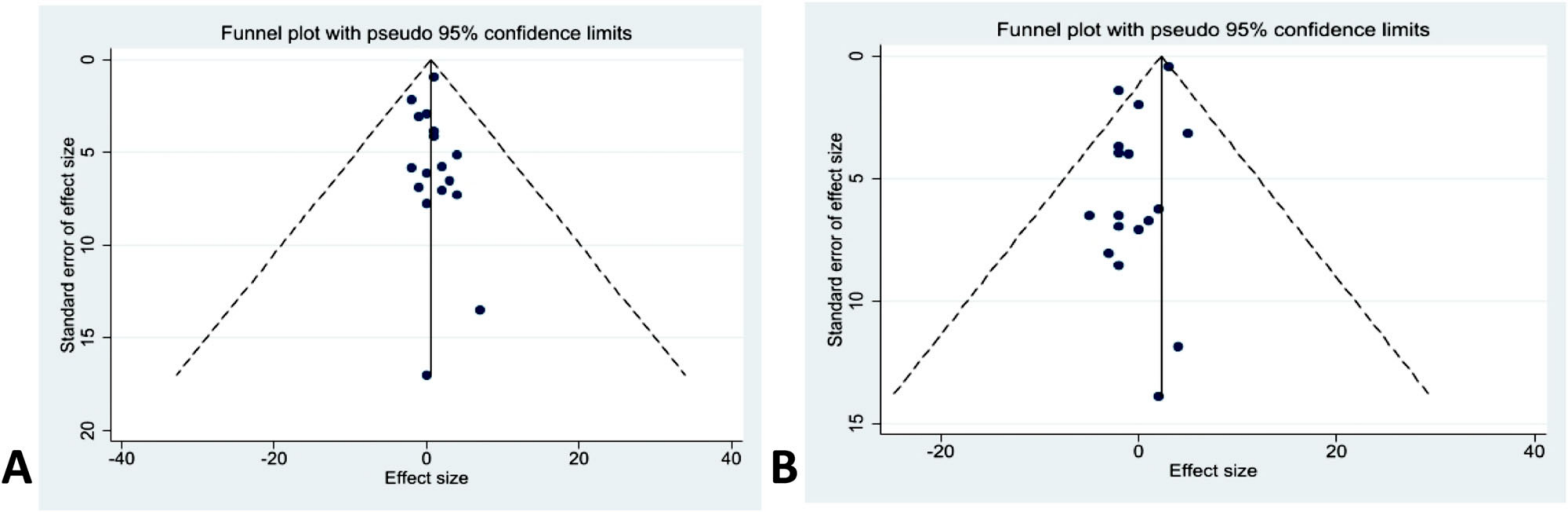
The funnel plot of the Montreal Cognitive Assessment and Mini‐Mental State Examination: (A) funnel plot of MoCA; (B) funnel plot of MMSE.

Funnel plot and Egger bias test findings are displayed in Figure 7. Tests for Egger bias for the prevalence of MoCA (*p* = 0.78, bias = 0.284; 95% CI, −0.31 to 0.41) indicated that there was no publication bias. But Egger bias tests for the incidence of MMSE (*p* = 0.017, bias = −2.69; 95% CI, −1.33 to −0.16) showed that publication bias was present. In terms of the robustness analysis of the review, the study takes a case‐by‐case elimination approach to demonstrate that the conclusions drawn in this study were documented. The results were plotted in the Supporting Information section.

### 3.9 Safety Evaluation

Totally two studies (Jiang et al. 2016; Sun 2013) reported adverse events. There were three adverse events in two groups (one in the study group and two in the controls) in Jiang et al. (2016) trial no longer appropriate to the trial, the incidence of which was 2% in the acupuncture group and 4% in the routine western treatment group. There was one adverse event in the treatment group in the trial of Sun (2013), namely, erythema and pruritus at the needle site, which disappeared after warm compression by means of a heated cloth followed by applying mint cream; the incidence of adverse events in the acupuncture group was 0.3%, and in the remaining trials, there were no adverse events.

## 4 Discussion

### 4.1 Summary and Interpretation

To the best of our knowledge, all the trials were published between 2011 and 2023, which shows that special attention has been given to finding out whether acupuncture works in recent years. Our results suggest that acupuncture improves cognitive function and activities of daily living with good safety. It was found that acupuncture was effective in patients with PSCI at week 4, but it was controversial at weeks 8 and 12. This study was compared with previously published systematic and meta‐analysis (Liu et al. 2014), and due to different inclusion and exclusion criteria, the intervention for the included articles in this article was acupuncture treatment, which is different from the articles included in this article but still useful for clinical research. Moreover, this article was compared with previously published systematic and meta‐analyses, and due to different inclusion and exclusion criteria, the intervention for the included articles in this article was acupuncture treatment, which is different from the articles included in this article but still useful for clinical research. Moreover, it is found that the control group of this article takes the drug Donepezil (Liu et al. 2016) unlike other articles that also use the drug as a control group, more nimodipine is used, and subsequent articles can be carried out.

### 4.2 Effect on Cognition

Overall, there were significantly higher MoCA scores among those in acupuncture than those in the controls, implying that acupuncture may represent a valid therapy for improving the cognition of PSCI survivors. Subgroup analysis was used to test whether different intervention methods of the eligible RCTs would lead to differences in MoCA. The acupuncture group versus medication was significant overall, apart from the sham acupuncture method, which may be due to small sample sizes causing bias. Conclusions in this study were in line with those of Kuang et al. (2021) reporting that although manual therapy for PSCI may be both efficacious and harmless, findings have to be cautious because of high Robs of trials enrolled plus a lack of quality of evidence for the endpoints evaluated. Similar to the above reviews, multiple methodological problems were encountered.

This study utilized subgroup analysis at various intervention dates for the MoCA score and came to the conclusion that acupuncture therapy was significant at weeks 4 and 12, but not statistically significant at week 8. The results of the forest plot data integration at week 8 indicated *p* = 0.06. This may be because patients’ cognitive performance gradually degrades with age or illness development. As a result, future research might expand the sample size to produce significant, excellent RCTs.

After doing a subgroup analysis with the timing of the intervention as a covariate, it was discovered that acupuncture was most helpful overall for the MMSE scores at week 4. Although week 12 was statistically significant for the MoCA score, the data did not demonstrate statistical significance at 12 weeks. This might be attributed to the results being biased by the fact that there was only one publication that contained data synthesis at 12 weeks, the dearth of high‐quality studies at the moment, and potential future proposals to include trials with lengthier treatments. Researchers might create future studies with an intervention length of at least 4 to further study the efficacy of various intervention durations and to offer empirical support for clinical practice.

It is important to note that the results of this meta‐analysis revealed that the MMSE and MoCA scores of stroke patients in the acupuncture group considerably improved after 4 weeks compared to the controls. However, the MMSE score was statistically significant at week 8, whereas the MoCA score was not. Kim et al. (2019) conducted a comparable systematic review, but the publication bias and differences in the precise acupoints used in the research were not conducted. However, the publication bias of MoCA and MMSE was investigated in this study. This indicated the necessity for additional thorough trials and a more rigorous investigation. This study demonstrated that acupuncture may be a complementary treatment for PSCI.

P300 ERP is an important neuroelectrophysiological index for the detection of cognitive function (Sun et al. 2017). According to studies, the decline in wave amplitude and increase in P300 latency are directly associated with the deterioration in cognitive performance (Toyoshima et al. 2020). The modification of P300 may reflect the efficiency of information encoding and processing by brain neurons as well as their capacity to recognize external inputs. The results of this investigation showed that acupuncture could significantly reduce the latency of P300 and lengthen its amplitude. Additionally, Zhan et al. (2017) evaluated 14 studies that showed acupuncture might enhance cognitive performance in PSCI patients. It was suggested that acupuncture might hasten the identification, evaluation, and encoding of environmental stimuli for information processing.

LOTCA, which was originally used to assess cognitive function in patients with PSCI, has been widely used in the assessment of various brain diseases, particularly cognitive dysfunction after cerebrovascular disease (Yao and Zou 2022; Zhang et al. 2022). This study indicated that acupuncture can improve the score of the LOTCA. A similar meta‐analysis also reported that acupuncture could increase the score of the LOTCA (Liu et al. 2023). Findings from the current meta‐analysis suggest that acupuncture could be an affordable approach for the recovery of cognitive function in patients with stroke.

Acupuncture has shown potential in enhancing cognitive function in individuals with PSCI. The underlying mechanism involves the capacity of acupuncture to augment hippocampal volume at the regional level and enhance the structural and functional connectivity of the hippocampus at the connectivity level. This, in turn, leads to improvements in cognitive function among patients with PSCI (Zhang et al. 2022). Moreover, acupuncture has been demonstrated to enhance cerebral blood flow and improve conditions in various cortical areas of the brain. It can significantly improve cognitive function and motor capabilities in patients with PSCI, and it is considered a promising therapeutic intervention (Fu et al. 2012; Wu et al. 2008). To evaluate the impact of acupuncture on PSCI patients, more high‐quality RCTs with a large sample size are required.

### 4.3 Effect on Daily Living

The results of this review showed statistically significant results in terms of BI scores, indicating that acupuncture was statistically significant in improving the living behavior of patients with PSCI. Contrarily, Zhan et al. (2017) reported that there was no significant difference in BI scores between the acupuncture and the controls in this review. It was indicated that acupuncture and other treatments may be equally effective in improving activities of daily living in patients with PSCI.

Similar to the conclusions drawn by Zhao's article (Zhao, Shang, and Zhao 2023), acupuncture appears to be effective in improving the quality of life of patients with PSCI. The potential mechanism could be that acupuncture enhances synaptic plasticity, thereby improving cognitive functions, which may assist patients in better adapting to and regaining their daily living skills. Another possible mechanism is that acupuncture modulates the concentration of neurotransmitters and metabolites in the brain, which could help improve attention, memory, and executive functions, thus enhancing the ability to perform daily activities. Additionally, acupuncture might indirectly affect the quality of life by regulating patients’ emotional and psychological states; for instance, reducing symptoms of anxiety and depression can improve cognitive capacity and overall well‐being (Tu, Lin, and Zhuang 2024).

### 4.4 Effect on Depression

The literature review conducted for this study revealed that acupuncture possesses efficacy in managing depressive symptoms, as indicated by recent research (Cai et al. 2019; Lam et al. 2023). The original design of the study was centered on cognitive function as the principal outcome of interest, with the result that depression was not initially considered in the search criteria. Nonetheless, during the analysis phase, a single study was identified that documented the concurrent influence of acupuncture on both depressive symptoms and cognitive performance.

Regarding post‐stroke depression, this study conducted a comprehensive review of the 21 selected articles. Among them, only one study investigated the impact of acupuncture on depression levels in patients with PSCI, yielding non‐statistically significant results (*p* = 0.89) (Huang et al. 2021). Specifically, our study primarily focused on cognitive function in patients with PSCI, which differs from the inclusion and exclusion criteria of other meta‐analyses. Additionally, the assessment scales used in our study are distinct, and this article notes that only one article reported on the depression status of PSCI patients, which may introduce a potential bias in our findings.

Additionally, this study has expanded discussion to include an analysis of the effects of acupuncture on cognition and depression, as well as a description of the relationship between depression and cognition. This indirectly provides suggestions for future research directions on the use of acupuncture to improve depression or cognitive function.

Nevertheless, evidence indicates a correlation between post‐stroke depression and cognitive function, highlighting the cognitive status of patients as a determining factor in post‐stroke depression (Su et al. 2023). Furthermore, individuals with post‐stroke depression exhibit more pronounced cognitive impairment and an elevated risk of mortality compared to post‐stroke patients without depression.

### 4.5 Acupuncture Point Selection and Effective Rate

The current study identified that the common acupoints in the eligible studies were GV20, GV24, EX‐HN1, PC6, SP6, ST36, and GB20. These acupoints have the effect of activating blood circulation, removing blood congestion, and excavating the qi machine. In this study, PC6 is one of the intersection points of eight veins (Liu et al. 2020), which has the function of nourishing the mind, calming the mind, and dredging qi and blood (Liu et al. 2020; Yang et al. 2022). The acupoint SP6, known as ‘Sanyinjiao’, serves to tonify the kidney and nourish Yin, thereby enriching the essence and promoting the production of marrow. Meanwhile, GB20, referred to as ‘Fengchi’, is effective in expelling wind, clearing the liver and gallbladder, dissipating cold, and alleviating pain. Additionally, it plays a role in soothing the meridians and collaterals. (Chang 2012).

The effective rate is an overall evaluation criterion. Efficacy assessment criteria: Efficacy was assessed using the theory [(score before treatment − score after treatment) ÷ score before treatment] × 100%. Remarkably effective: ≥70%; effective: ≥30% and <70%; ineffective: <30%; worsening: ≥−20% (Hu et al. 2016; Tian 2002). The score refers to the pre‐treatment TCM symptom score and the post‐treatment TCM symptom score (Tian 2002). A systematic review conducted in 2023 by Liu et al. (2023) came to the conclusion that the underlying studies were comparable in that acupuncture generally improved cognitive function in PSCI patients. This result provided a pertinent clinical indication and reference that acupuncture may be effective for improving cognitive function in PSCI patients.

### 4.6 Strengths and Limitations

This study has several strengths. This review's exhaustive systematic search ensured the inclusion of all relevant and large‐scale studies, reducing the risk of publication bias compared with previous reviews. Moreover, the review built upon previous research by placing a greater emphasis on the duration of intervention. This review has identified a gap in the literature regarding the long‐term effects and sustainability of interventions. Using sensitivity analyses to confirm the robustness of the results and meta‐regression and Egger tests to improve statistical ability, we ensured that our meta‐analysis provided practical evidence of high relevance to caregivers and clinicians. There are still some limitations to this study; language constraints resulted in this study only reviewing original articles in English and Chinese, followed by the overall poor quality of the included articles, which resulted in an ending bias.

### 4.7 Expectation

On the basis of the results of this study, the following issues should be addressed to improve the methodological quality of clinical trials in the future. (1) The randomization procedures, allocation concealment, and blinding methods should be clearly described and fully reported. (2) Withdrawals/Dropouts and adverse events in trials should be clearly reported. (3) Diagnosis and adjudication criteria should be consistent and specified. (4) All clinical trials should be registered in advance, and published articles should include links to the protocol. (5) Further studies on the long‐term effects of acupuncture should be carried out in the future in this area of research.

## 5 Conclusion

In conclusion, the study contributes to the growing body of evidence supporting the role of acupuncture in the neurorehabilitation of PSCI patients. The observed benefits at 4 and 8 weeks highlight the potential for acupuncture to serve as a valuable adjunct to conventional therapies. As we continue to explore the complexities of PSCI and its management, the integration of traditional and modern medicine, such as acupuncture, holds great promise for ameliorating cognitive deficits and enhancing daily functional abilities.

## 6 Relevance to Clinical Practice

Acupuncture may help patients with PSCI recover by improving their cognitive function. From the standpoint of medical professionals, this study can enlighten clinical workers in practical clinical work, who can select appropriate acupoints according to the actual conditions of patients, as well as confirm the treatment course of PSCI patients, while paying attention to observing and evaluating the therapeutic efficacy of acupuncture, to improve the health outcomes of patients in a patient‐centered way.

## Author Contributions

**Ziyan Luo**: writing–original draft, data curation. **Wenxuan Li**: data curation. **Jieting Jiang**: data curation. **Jie Sun**: data curation. **Mingyue Zhang**: visualization. **Yaqing Zhang**: supervision, methodology. **Lu Dong**: supervision, methodology. **Kunpeng Li**: methodology, supervision. **Caiqin Wu**: funding acquisition, writing–review and editing, supervision, methodology.

## Conflicts of Interest

The authors declare no conflicts of interest.

### Peer Review

The peer review history for this article is available at https://publons.com/publon/10.1002/brb3.70075.

## Supporting information



Additional supporting information can be found online in the Supporting Information section.

## Data Availability

All the datasets of the current study are available from the corresponding author on reasonable request.
