# Supplementary material for: Effect of Acupuncture on Cognitive Function in Patients With Post‐Stroke Cognitive Impairment: A Systematic Review and Meta‐Analysis
Source: Brain Behav. 2024 Oct 14;14(10):e70075. doi: 10.1002/brb3.70075 (PMC11473547; doi:10.1002/brb3.70075)
Supplement: Supplementary file 1 — Additional supporting information can be found online in the Supporting Information section. [file BRB3-14-e70075-s001.docx]

**Supplementary eMethod 1: Search strategy of each database**

**Cochrane Library**

#1 MeSH descriptor: [Stroke] explode all trees

#2 (Apoplexy):ti,ab,kw OR (Cerebral stroke):ti,ab,kw OR (Cerebrovascular disease):ti,ab,kw OR (Cerebrovascular accident):ti,ab,kw OR (Cerebrovascular disorders):ti,ab,kw (Word variations have been searched)

#3 (Cerebral hemorrhage):ti,ab,kw OR (Hemorrhagic apoplexy):ti,ab,kw OR (hemorrhagic stroke):ti,ab,kw OR (Cerebral infarction):ti,ab,kw OR (Brain infarction):ti,ab,kw (Word variations have been searched)

#4 (Ischemic stroke):ti,ab,kw OR (Cerebrovascular Stroke):ti,ab,kw OR (CVA):ti,ab,kw OR (Brain Vascular Accident):ti,ab,kw OR (Acute Stroke):ti,ab,kw (Word variations have been searched)

#5 MeSH descriptor: [Cognition] explode all trees

#6 (Cognitive Dysfunction):ti,ab,kw OR (Cognitive Impairments):ti,ab,kw OR (Mild Cognitive Impairment):ti,ab,kw OR (Mild Neurocognitive Disorder):ti,ab,kw OR (Cognitive Decline):ti,ab,kw (Word variations have been searched)

#7 (Mental Deterioration):ti,ab,kw OR (Cognitive deficit):ti,ab,kw OR (PSCI):ti,ab,kw (Word variations have been searched)

#8 #5 or #6 or #7

#9 MeSH descriptor: [Acupuncture] explode all trees

#10 (acupuncture point):ti,ab,kw OR (acupuncture therapy):ti,ab,kw OR (acupuncture analgesia):ti,ab,kw (Word variations have been searched)

#11 #9 or #10

#12 (Acute Cerebrovascular Accident):ti,ab,kw OR (Cerebrovascular Apoplexy):ti,ab,kw (Word variations have been searched)

#13 #1 or #2 or #3 or #4 or #12

#14 #8 AND #11 AND #13

**PubMed (using MeSH term)**

(("cognition"[MeSH Terms] OR "cognitive dysfunction"[MeSH Terms] OR "cognition disorders"[MeSH Terms]) AND "stroke"[MeSH Terms] AND ("acupuncture"[MeSH Terms] OR "acupuncture therapy"[MeSH Terms])) AND (clinicaltrial[Filter] OR randomizedcontrolledtrial[Filter])

**EMBASE (using Emtree term in the PICO search function)**

('cerebrovascular accident'/exp OR 'stroke':ti,ab OR 'cva':ti,ab OR 'cerebrovascular apoplexy':ti,ab OR 'brain vascular accident':ti,ab OR 'cerebrovascular stroke':ti,ab OR 'cerebrovascular strokes':ti,ab OR 'cvas':ti,ab OR 'strokes':ti,ab OR 'cerebrovascular accidents':ti,ab OR 'apoplexy, cerebrovascular':ti,ab OR 'vascular accident, brain':ti,ab OR 'brain vascular accident':ti,ab OR 'brain vascular accidents':ti,ab OR 'stroke, cerebrovascular':ti,ab OR 'strokes, cerebrovascular':ti,ab OR 'apoplexy':ti,ab OR 'cerebral stroke':ti,ab OR 'cerebral strokes':ti,ab OR 'stroke, cerebral':ti,ab OR 'strokes, cerebral':ti,ab OR 'stroke, acute':ti,ab OR 'strokes, acute':ti,ab OR 'acute strokes':ti,ab OR 'acute stroke':ti,ab OR 'cerebrovascular accident, acute':ti,ab OR 'cerebrovascular accidents, acute':ti,ab OR 'acute cerebrovascular accidents':ti,ab OR 'acute cerebrovascular accident':ti,ab) AND ('cognition'/exp OR 'cognitive dysfunction':ab,ti OR 'cognitive dysfunctions':ab,ti OR 'dysfunction, cognitive':ab,ti OR 'dysfunctions, cognitive':ab,ti OR 'cognitive impairments':ab,ti OR 'cognitive impairment':ab,ti OR 'impairment, cognitive':ab,ti OR 'impairments, cognitive':ab,ti OR 'mild cognitive impairment':ab,ti OR 'mild cognitive impairments':ab,ti OR 'cognitive impairment, mild':ab,ti OR 'cognitive impairments, mild':ab,ti OR 'impairment, mild cognitive':ab,ti OR 'impairment, mild cognitive':ab,ti OR 'impairments, mild cognitive':ab,ti OR 'mild neurocognitive disorder':ab,ti OR 'mild neurocognitive disorders':ab,ti OR 'disorders, mild neurocognitive':ab,ti OR 'disorder, mild neurocognitive':ab,ti OR 'neurocognitive disorders, mild':ab,ti OR 'neurocognitive disorder, mild':ab,ti OR 'cognitive decline':ab,ti OR 'cognitive declines':ab,ti OR 'declines, cognitive':ab,ti OR 'decline, cognitive':ab,ti OR 'deterioration, mental':ab,ti OR 'deteriorations, mental':ab,ti OR 'mental deteriorations':ab,ti OR 'mental deterioration':ab,ti) AND ('random':ti,ab OR 'placebo':ti,ab OR 'double-blind':ti,ab) AND ('acupuncture'/exp OR 'acupuncture point':ti,ab OR 'acupuncture therapy':ti,ab OR 'acupuncture points':ti,ab OR 'acupuncture analgesia':ti,ab)

**Scopus**

( TITLE-ABS-KEY ( stroke OR cva ) AND TITLE-ABS-KEY ( cognition OR cognitivedysfunction ) AND TITLE-ABS-KEY ( acupuncture ) AND TITLE-ABS-KEY ( randomizedcontrolledtrial OR randomized OR placebo ) )

**Ebsco**


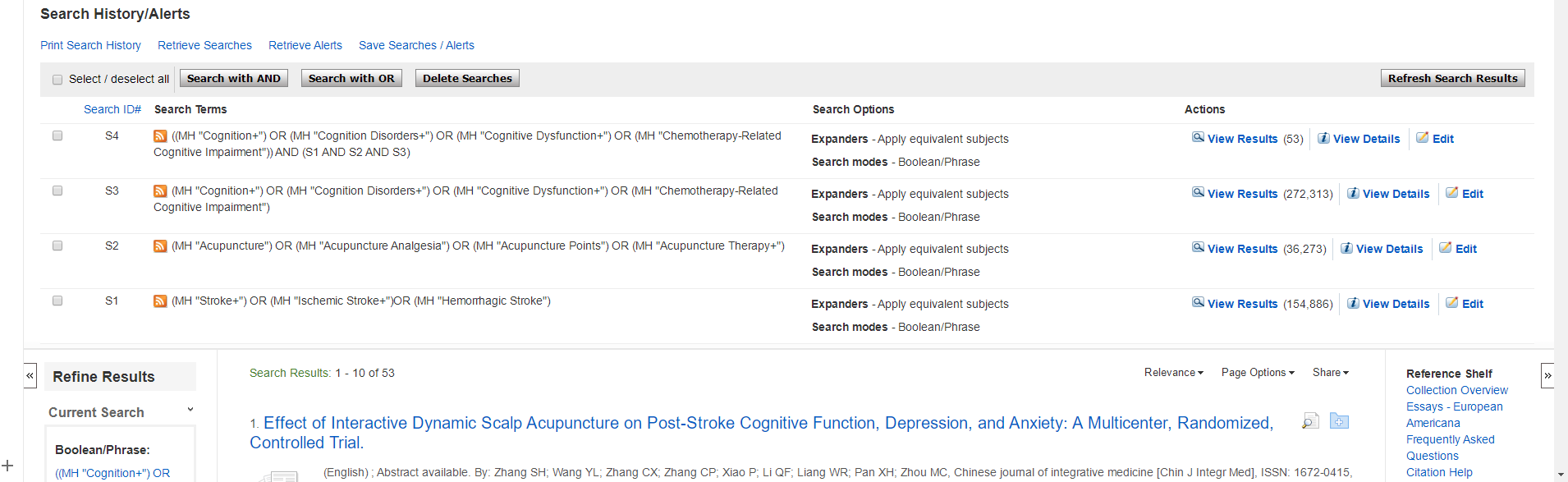


**Web of Science**


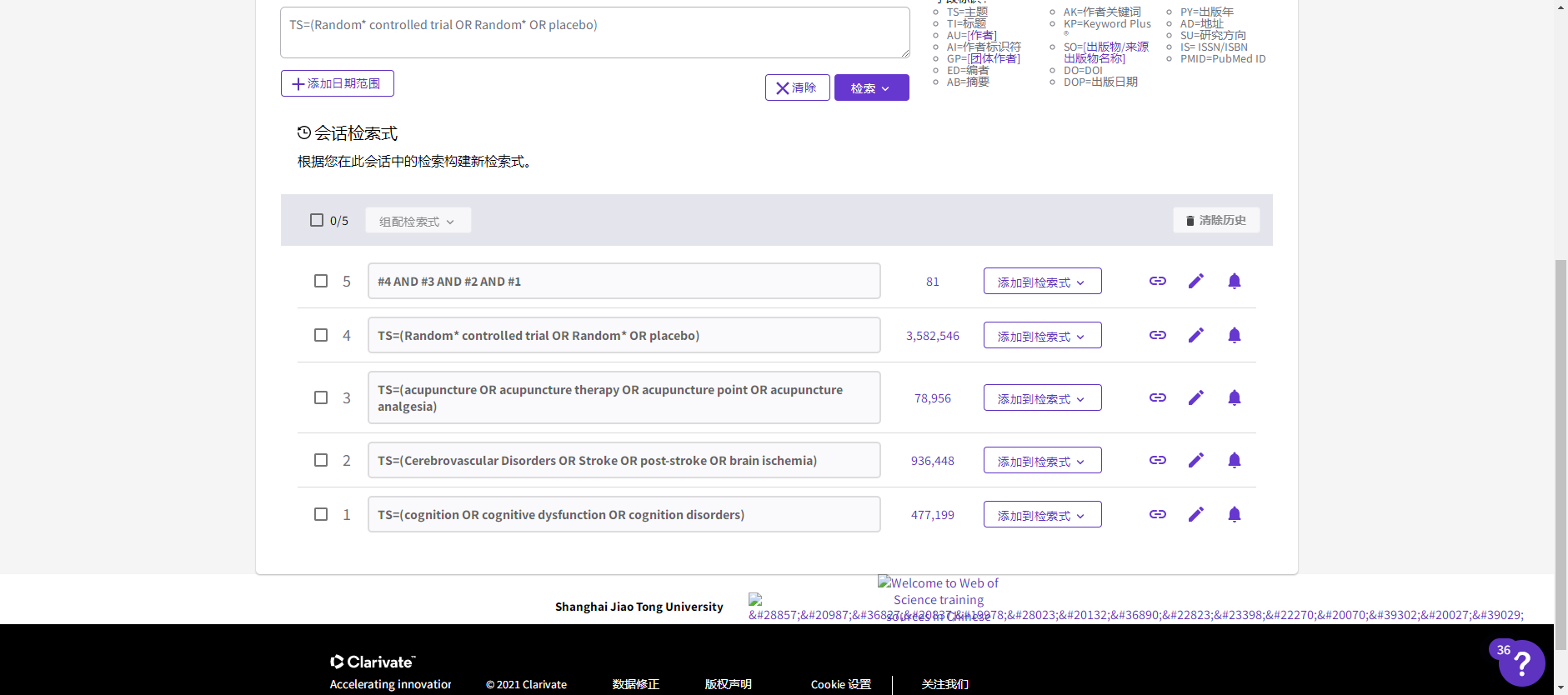


**China National Knowledge Infrastructure, CNKI：**

|  | **Strategy** | **Number** |
| --- | --- | --- |
| **1（P）** | [(SU=naocuzhong) OR (SU=zhongfeng) OR (SU=naoxueguanzhangai) OR (SU=naogengsi) OR (SU=naoshuansai) OR(SU=naochuxue) OR (SU=naogengsai)](https://kns.cnki.net/kns8/AdvSearch?id=110&dbcode=CFLS&searchtype=gradeSearch&ishistory=1" \t "https://kns.cnki.net/kns8/manage/_blank" \o "(关键词=八段锦) OR (关键词=太极拳) OR (关键词=五禽戏) OR (关键词=六字诀) OR (关键词=气功) OR (关键词=易筋经) OR (关键词=五行掌) OR (关键词=站桩功) OR (关键词=回春功)) |  |
| **2（I）** | [(SU=zhenjiu) OR (SU=zhenci)OR (SU=touzhen)OR (SU=dianzhen)](https://kns.cnki.net/kns8/AdvSearch?id=110&dbcode=CFLS&searchtype=gradeSearch&ishistory=1" \t "https://kns.cnki.net/kns8/manage/_blank" \o "(关键词=八段锦) OR (关键词=太极拳) OR (关键词=五禽戏) OR (关键词=六字诀) OR (关键词=气功) OR (关键词=易筋经) OR (关键词=五行掌) OR (关键词=站桩功) OR (关键词=回春功))OR (SU=tizhen) |  |
| **3（O）** | [(SU=renzhenzhangai) OR (SU=renzhigongnengzhangai)](https://kns.cnki.net/kns8/AdvSearch?id=110&dbcode=CFLS&searchtype=gradeSearch&ishistory=1" \t "https://kns.cnki.net/kns8/manage/_blank" \o "(关键词=八段锦) OR (关键词=太极拳) OR (关键词=五禽戏) OR (关键词=六字诀) OR (关键词=气功) OR (关键词=易筋经) OR (关键词=五行掌) OR (关键词=站桩功) OR (关键词=回春功)) |  |
| **4** | **(**AB=suijiduizhao) OR (AB=suiji) OR (AB=RCT) |  |
| **5** | **#1 AND #2 AND #3 AND #4** | **50** |

**VIP：**

|  | **Strategy** | **Number** |
| --- | --- | --- |
| **1（P）** | [(SU=naocuzhong) OR (SU=zhongfeng) OR (SU=naoxueguanzhangai) OR (SU=naogengsi) OR (SU=naoshuansai) OR(SU=naochuxue) OR (SU=naogengsai)](https://kns.cnki.net/kns8/AdvSearch?id=110&dbcode=CFLS&searchtype=gradeSearch&ishistory=1" \t "https://kns.cnki.net/kns8/manage/_blank" \o "(关键词=八段锦) OR (关键词=太极拳) OR (关键词=五禽戏) OR (关键词=六字诀) OR (关键词=气功) OR (关键词=易筋经) OR (关键词=五行掌) OR (关键词=站桩功) OR (关键词=回春功)) |  |
| **2（I）** | [(SU=zhenjiu) OR (SU=zhenci)OR (SU=touzhen)OR (SU=dianzhen)](https://kns.cnki.net/kns8/AdvSearch?id=110&dbcode=CFLS&searchtype=gradeSearch&ishistory=1" \t "https://kns.cnki.net/kns8/manage/_blank" \o "(关键词=八段锦) OR (关键词=太极拳) OR (关键词=五禽戏) OR (关键词=六字诀) OR (关键词=气功) OR (关键词=易筋经) OR (关键词=五行掌) OR (关键词=站桩功) OR (关键词=回春功))OR (SU=tizhen) |  |
| **3（O）** | [((SU=renzhenzhangai) OR (SU=renzhigongnengzhangai)](https://kns.cnki.net/kns8/AdvSearch?id=110&dbcode=CFLS&searchtype=gradeSearch&ishistory=1" \t "https://kns.cnki.net/kns8/manage/_blank" \o "(关键词=八段锦) OR (关键词=太极拳) OR (关键词=五禽戏) OR (关键词=六字诀) OR (关键词=气功) OR (关键词=易筋经) OR (关键词=五行掌) OR (关键词=站桩功) OR (关键词=回春功)) |  |
| **4** | **(**AB=suijiduizhao) OR (AB=suiji) OR (AB=RCT) |  |
| **5** | **#1 AND #2 AND #3 AND #4** | **11** |

**Wanfang Data：**

| **1（P）** | [(SU=naocuzhong) OR (SU=zhongfeng) OR (SU=naoxueguanzhangai) OR (SU=naogengsi) OR (SU=naoshuansai) OR(SU=naochuxue) OR (SU=naogengsai)](https://kns.cnki.net/kns8/AdvSearch?id=110&dbcode=CFLS&searchtype=gradeSearch&ishistory=1" \t "https://kns.cnki.net/kns8/manage/_blank" \o "(关键词=八段锦) OR (关键词=太极拳) OR (关键词=五禽戏) OR (关键词=六字诀) OR (关键词=气功) OR (关键词=易筋经) OR (关键词=五行掌) OR (关键词=站桩功) OR (关键词=回春功)) |  |
| --- | --- | --- |
| **2（I）** | [(SU=zhenjiu) OR (SU=zhenci)OR (SU=touzhen)OR (SU=dianzhen)](https://kns.cnki.net/kns8/AdvSearch?id=110&dbcode=CFLS&searchtype=gradeSearch&ishistory=1" \t "https://kns.cnki.net/kns8/manage/_blank" \o "(关键词=八段锦) OR (关键词=太极拳) OR (关键词=五禽戏) OR (关键词=六字诀) OR (关键词=气功) OR (关键词=易筋经) OR (关键词=五行掌) OR (关键词=站桩功) OR (关键词=回春功))OR (SU=tizhen) |  |
| **3（O）** | [((SU=renzhenzhangai) OR (SU=renzhigongnengzhangai)](https://kns.cnki.net/kns8/AdvSearch?id=110&dbcode=CFLS&searchtype=gradeSearch&ishistory=1" \t "https://kns.cnki.net/kns8/manage/_blank" \o "(关键词=八段锦) OR (关键词=太极拳) OR (关键词=五禽戏) OR (关键词=六字诀) OR (关键词=气功) OR (关键词=易筋经) OR (关键词=五行掌) OR (关键词=站桩功) OR (关键词=回春功)) |  |
| **4** | **(**AB=suijiduizhao) OR (AB=suiji) OR (AB=RCT) |  |
| **5** | **#1 AND #2 AND #3 AND #4** | **107** |

**Table 1 – PRISMA checklist 2020**

| **Section and Topic** | **Item #** | **Checklist item** | **Location where item is reported** |
| --- | --- | --- | --- |
| **TITLE** | | |  |
| Title | 1 | Identify the report as a systematic review. | Page 1, line 1~2 |
| **ABSTRACT** | | |  |
| Abstract | 2 | See the PRISMA 2020 for Abstracts checklist. | Page 10 to 11, line 115 to 119 |
| **INTRODUCTION** | | |  |
| Rationale | 3 | Describe the rationale for the review in the context of existing knowledge. | Page 9 to 10, line 52~102 |
| Objectives | 4 | Provide an explicit statement of the objective(s) or question(s) the review addresses. | Page10, line 103~112 |
| **METHODS** | | |  |
| Eligibility criteria | 5 | Specify the inclusion and exclusion criteria for the review and how studies were grouped for the syntheses. | Page12, line 145~160 |
| Information sources | 6 | Specify all databases, registers, websites, organisations, reference lists and other sources searched or consulted to identify studies. Specify the date when each source was last searched or consulted. | Page 11, line 122~126 |
| Search strategy | 7 | Present the full search strategies for all databases, registers and websites, including any filters and limits used. | Page 11, line 126~133 and supplementary files |
| Selection process | 8 | Specify the methods used to decide whether a study met the inclusion criteria of the review, including how many reviewers screened each record and each report retrieved, whether they worked independently, and if applicable, details of automation tools used in the process. | Page 11, line 137~138 |
| Data collection process | 9 | Specify the methods used to collect data from reports, including how many reviewers collected data from each report, whether they worked independently, any processes for obtaining or confirming data from study investigators, and if applicable, details of automation tools used in the process. | Page 13, line 174~175 |
| Data items | 10a | List and define all outcomes for which data were sought. Specify whether all results that were compatible with each outcome domain in each study were sought (e.g. for all measures, time points, analyses), and if not, the methods used to decide which results to collect. | Page 12, line 146~160 |
|  | 10b | List and define all other variables for which data were sought (e.g. participant and intervention characteristics, funding sources). Describe any assumptions made about any missing or unclear information. | Page 12, line 150~160 |
| Study risk of bias assessment | 11 | Specify the methods used to assess risk of bias in the included studies, including details of the tool(s) used, how many reviewers assessed each study and whether they worked independently, and if applicable, details of automation tools used in the process. | Page 14, line 183~198 |
| Effect measures | 12 | Specify for each outcome the effect measure(s) (e.g. risk ratio, mean difference) used in the synthesis or presentation of results. | Page 14 to 15, line 201~219 |
| Synthesis methods | 13a | Describe the processes used to decide which studies were eligible for each synthesis (e.g. tabulating the study intervention characteristics and comparing against the planned groups for each synthesis (item #5)). | Page 14 to 15, line 201~219 |
|  | 13b | Describe any methods required to prepare the data for presentation or synthesis, such as handling of missing summary statistics, or data conversions. | Page 14 to 15, line 201~219 |
|  | 13c | Describe any methods used to tabulate or visually display results of individual studies and syntheses. | Page 15, line 214~219 |
|  | 13d | Describe any methods used to synthesize results and provide a rationale for the choice(s). If meta-analysis was performed, describe the model(s), method(s) to identify the presence and extent of statistical heterogeneity, and software package(s) used. | Page 15, line 216~219 |
|  | 13e | Describe any methods used to explore possible causes of heterogeneity among study results (e.g. subgroup analysis, meta-regression). | Page 15, line 216~219 |
|  | 13f | Describe any sensitivity analyses conducted to assess robustness of the synthesized results. | Page 15, line 216~219 |
| Reporting bias assessment | 14 | Describe any methods used to assess risk of bias due to missing results in a synthesis (arising from reporting biases). | Page 14 to 15, line 201~219 |
| Certainty assessment | 15 | Describe any methods used to assess certainty (or confidence) in the body of evidence for an outcome. | Page 14 to 15, line 201~219 |
| **RESULTS** | | |  |
| Study selection | 16a | Describe the results of the search and selection process, from the number of records identified in the search to the number of studies included in the review, ideally using a flow diagram. | Page 15 to 16, line 223~237; Figure 1. |
|  | 16b | Cite studies that might appear to meet the inclusion criteria, but which were excluded, and explain why they were excluded. | Page 15 to 16, line 223~237 |
| Study characteristics | 17 | Cite each included study and present its characteristics. | Page 16 to 17, line 240~263; Table 1 |
| Risk of bias in studies | 18 | Present assessments of risk of bias for each included study. | Page 17 to 18, line 265~281; Figure 2. |
| Results of individual studies | 19 | For all outcomes, present, for each study: (a) summary statistics for each group (where appropriate) and (b) an effect estimate and its precision (e.g. confidence/credible interval), ideally using structured tables or plots. | Page 18 to 22, Figure 3 to Figure 6 |
| Results of syntheses | 20a | For each synthesis, briefly summarise the characteristics and risk of bias among contributing studies. | Page 18 to 22, Figure 3 to Figure 6 |
|  | 20b | Present results of all statistical syntheses conducted. If meta-analysis was done, present for each the summary estimate and its precision (e.g. confidence/credible interval) and measures of statistical heterogeneity. If comparing groups, describe the direction of the effect. | Page 18 to 22, Figure 3 to Figure 6 |
|  | 20c | Present results of all investigations of possible causes of heterogeneity among study results. | Page 18 to 22, Figure 3 to Figure 6 |
|  | 20d | Present results of all sensitivity analyses conducted to assess the robustness of the synthesized results. | Page 21 to 22, line 377~384; supplementary files |
| Reporting biases | 21 | Present assessments of risk of bias due to missing results (arising from reporting biases) for each synthesis assessed. | Page 21 to 22, line 377~384; Figure 7 |
| Certainty of evidence | 22 | Present assessments of certainty (or confidence) in the body of evidence for each outcome assessed. | Page 21 to 22, line 377~384; Figure 7 |
| **DISCUSSION** | | |  |
| Discussion | 23a | Provide a general interpretation of the results in the context of other evidence. | Page 23 to 24, line 398~416 |
|  | 23b | Discuss any limitations of the evidence included in the review. | Page 31 to 32, line 567~579 |
|  | 23c | Discuss any limitations of the review processes used. | Page 31 to 32, line 567~579 |
|  | 23d | Discuss implications of the results for practice, policy, and future research. | Page 33, line 602~609 |
| **OTHER INFORMATION** | | |  |
| Registration and protocol | 24a | Provide registration information for the review, including register name and registration number, or state that the review was not registered. | Page 9 to 10, line 115~119 |
|  | 24b | Indicate where the review protocol can be accessed, or state that a protocol was not prepared. | Title page |
|  | 24c | Describe and explain any amendments to information provided at registration or in the protocol. | N/A |
| Support | 25 | Describe sources of financial or non-financial support for the review, and the role of the funders or sponsors in the review. | Title page |
| Competing interests | 26 | Declare any competing interests of review authors. | Title page |
| Availability of data, code and other materials | 27 | Report which of the following are publicly available and where they can be found: template data collection forms; data extracted from included studies; data used for all analyses; analytic code; any other materials used in the review. | Supplementary flies |

*From:*  Page MJ, McKenzie JE, Bossuyt PM, Boutron I, Hoffmann TC, Mulrow CD, et al. The PRISMA 2020 statement: an updated guideline for reporting systematic reviews. BMJ 2021;372:n71. doi: 10.1136/bmj.n71

**eTable 1– PRISMA Abstract checklist 2020**

| **Section and Topic** | **Item #** | **Checklist item** | **Reported (Yes/No)** |
| --- | --- | --- | --- |
| **TITLE** | | |  |
| Title | 1 | Identify the report as a systematic review. | Yes |
| **BACKGROUND** | | |  |
| Objectives | 2 | Provide an explicit statement of the main objective(s) or question(s) the review addresses. | Yes |
| **METHODS** | | |  |
| Eligibility criteria | 3 | Specify the inclusion and exclusion criteria for the review. | Yes |
| Information sources | 4 | Specify the information sources (e.g. databases, registers) used to identify studies and the date when each was last searched.` | Yes |
| Risk of bias | 5 | Specify the methods used to assess risk of bias in the included studies. | Yes |
| Synthesis of results | 6 | Specify the methods used to present and synthesise results. | Yes |
| **RESULTS** | | |  |
| Included studies | 7 | Give the total number of included studies and participants and summarise relevant characteristics of studies. | Yes |
| Synthesis of results | 8 | Present results for main outcomes, preferably indicating the number of included studies and participants for each. If meta-analysis was done, report the summary estimate and confidence/credible interval. If comparing groups, indicate the direction of the effect (i.e. which group is favoured). | Yes |
| **DISCUSSION** | | |  |
| Limitations of evidence | 9 | Provide a brief summary of the limitations of the evidence included in the review (e.g. study risk of bias, inconsistency and imprecision). | Yes |
| Interpretation | 10 | Provide a general interpretation of the results and important implications. | Yes |
| **OTHER** | | |  |
| Funding | 11 | Specify the primary source of funding for the review. | Yes |
| Registration | 12 | Provide the register name and registration number. | Yes |

*From:*  Page MJ, McKenzie JE, Bossuyt PM, Boutron I, Hoffmann TC, Mulrow CD, et al. The PRISMA 2020 statement: an updated guideline for reporting systematic reviews. BMJ 2021;372:n71. doi: 10.1136/bmj.n71

**Table 2 – Risk of Bias Assessment**

| **Study** | **Overall**  **Assessment** | **Sequence**  **Generation** | **Allocation Concealment** | **Blinding:**  **Performance**  **Bias** | **Blinding:**  **Detection Bias** | **Incomplete**  **Outcome Data** | **Selective**  **Outcome**  **Reporting** | **Other Sources**  **of Bias** |
| --- | --- | --- | --- | --- | --- | --- | --- | --- |
| **Jing CHEN**  **2020** |  | The patients were randomly divided into a reference group and a  treatment group, each with 28 patients. | Not reported | Participants and study personnel  were aware of the study-group  assignments | Cognitive function was assessed with the single-blind method. People in charge of efficacy evaluation did not  know the grouping information of the patients. | All analyses by  intension to  treat.  Participants lost  to follow-up  documented  with reason (ie death) if  possible. | Pre-specified  outcomes  reported | Not reported |
| **Risk of Bias** | UNCLEAR | Low | Unclear | Unclear | Low | Low | Low | Unclear |
| **Cai JIANG**  **2016** |  | This was a randomized controlled trial with a 2  2 factorial design | Not reported | Participants and study personnel  were aware of the study-group  assignments | Not reported | Assumed  complete  outcome data  for all patients  as primary  outcome was  investigator  reported. | Pre-specified  outcomes  reported | Not reported |
| **Risk of Bias** | UNCLEAR | Low | Unclear | Unclear | Unclear | Low | Low | Unclear |
| **Li-fang CHEN**  **2016** |  | Randomization was computer-generated  by independent research staff using software, | The  generated list of random numbers was placed into sequentially numbered, opaque, sealed envelopes. | Participants and study personnel  were aware of the study-group  assignments | All  of the allopathic medical staff, rehabilitation therapists,  outcome assessors, and data analysts were blinded to  group assignments. | Assumed  complete  outcome data  for all patients  as primary  outcome was  investigator  reported. | Pre-specified  outcomes  reported | Not reported |
| **Risk of Bias** | UNCLEAR | Low | Low | Unclear | Low | Low | Low | Unclear |
| **Shu-hua WANG**  **2016** |  | The patient allocation  sequence was generated using a complete randomization  procedure. | The random number and the associated  allocated treatment were kept in sequentially numbered  sealed opaque envelopes. | Participants and study personnel  were aware of the study-group  assignments | Both the trained assessor, who  evaluated patient MoCA score, and the statistician, who  conducted the data analysis, were blinded to the treatment  allocation. | Amount of  missing  outcome data  was balanced  between the  treatment arms | Pre-specified  outcomes  reported | Not reported |
| **Risk of Bias** | UNCLEAR | Low | Low | Unclear | Low | Low | Low | Unclear |
| **Jian XIONG**  **2020** |  | This was a randomized, double-blinded clinical  trial with two parallel arms. | Not reported | Patients in the experimental group additionally received scalp acupuncture and cognitive training, while the  control group received sham scalp acupuncture and cognitive training. | The assessors and statisticians  were unaware of the group allocation throughout the  study period | All analyses by  intension to  treat.  Participants lost  to follow-up  documented  with reason (ie death) if  possible. | Pre-specified  outcomes  reported | Not reported |
| **Risk of Bias** | UNCLEAR | Low | Unclear | Low | Low | Low | Low | Unclear |
| **Fan YANG**  **2017** |  | A total of 60 eligible patients with post-stroke cognitive impairment were randomized into a treatment group and  a control group by random digital table. | Not reported | Participants and study personnel  were aware of the study-group  assignments | Not reported | Amount of  missing  outcome data  was balanced  between the  treatment arms | Pre-specified  outcomes  reported | Not reported |
| **Risk of Bias** | UNCLEAR | Low | Unclear | Unclear | Unclear | Low | Low | Unclear |
| **Rui YAO**  **2020** |  | According to the method of random number table, they  were divided into a treatment group and a control  group | Not reported | Participants and study personnel  were aware of the study-group  assignments | Not reported | All analyses by  intension to  treat.  Participants lost  to follow-up  documented  with reason (ie death) if  possible. | Pre-specified  outcomes  reported | Not reported |
| **Risk of Bias** | UNCLEAR | Low | Unclear | Unclear | Unclear | Low | Low | Unclear |
| **Li HUANG**  **2021** |  | A total of 120 eligible participants were randomly  assigned to receive either EA or sham SA via block  random method at a ratio of 1:1 | Acupuncturists  obtained each patient’s group assignment through an  opaque envelope from another researcher before preforming the intervention | For patients in SA group, a blunt-tipped placebo  needle provided participant-blinding effects with a  similar appearance to conventional needles | Not reported | Assumed  complete  outcome data  for all patients  as primary  outcome was  investigator  reported. | Pre-specified  outcomes  reported | Not reported |
| **Risk of Bias** | UNCLEAR | Low | Low | Low | Unclear | Low | Low | Unclear |
| **Li-tai SUN**  **2013** |  | According to the method of random number table, they  were divided into a treatment group and a control  group | Not reported | Participants and study personnel  were aware of the study-group  assignments | Not reported | Did not fully report the data mentioned | Pre-specified  outcomes  reported | Not reported |
| **Risk of Bias** | HIGH | Low | Unclear | Unclear | Unclear | High | Low | Unclear |
| **Xiao-dong FENG**  **2013** |  | According to the method of random number table, they  were divided into a treatment group and a control  group | Not reported | Participants and study personnel  were aware of the study-group  assignments | Not reported | Did not fully report the data mentioned | Pre-specified  outcomes  reported | Not reported |
| **Risk of Bias** | HIGH | Low | Unclear | Unclear | Unclear | High | Low | Unclear |
| **Lin LIU**  **2015** |  | According to the method of random number table, they  were divided into a treatment group and a control  group | The random number and the associated  allocated treatment were kept in sequentially numbered  sealed opaque envelopes. | Participants and study personnel  were aware of the study-group  assignments | Not reported | Assumed  complete  outcome data  for all patients  as primary  outcome was  investigator  reported. | Pre-specified  outcomes  reported | Not reported |
| **Risk of Bias** | UNCLEAR | Low | Low | Unclear | Unclear | Low | Low | Unclear |
| **Jia-jun KANG**  **2011** |  | According to the method of random number table, they  were divided into a treatment group and a control  group | The random number and the associated  allocated treatment were kept in sequentially numbered  sealed opaque envelopes. | Placebo  needle provided participant-blinding effects with a  similar appearance to conventional needles | The assessors and statisticians  were unaware of the group allocation throughout the  study period | All analyses by  intension to  treat.  Participants lost  to follow-up  documented  with reason (ie death) if  possible. | Pre-specified  outcomes  reported | Not reported |
| **Risk of Bias** | UNCLEAR | Low | Low | Low | Low | Low | Low | Unclear |
| **Ya-nan ZHANG**  **2014** |  | According to the method of random number table, they  were divided into a treatment group and a control  group | Not reported | Participants and study personnel  were aware of the study-group  assignments | Not reported | All analyses by  intension to  treat.  Participants lost  to follow-up  documented  with reason (ie death) if  possible. | Pre-specified  outcomes  reported | Not reported |
| **Risk of Bias** | UNCLEAR | Low | Unclear | Unclear | Unclear | Low | Low | Unclear |
| **Er-mei CAO**  **2015** |  | According to the method of random number table, they  were divided into a treatment group and a control  group | The random number and the associated  allocated treatment were kept in sequentially numbered  sealed opaque envelopes. | Participants and study personnel  were aware of the study-group  assignments | Not reported | All analyses by  intension to  treat.  Participants lost  to follow-up  documented  with reason (ie death) if  possible. | Pre-specified  outcomes  reported | Not reported |
| **Risk of Bias** | UNCLEAR | Low | Low | Unclear | Unclear | Low | Low | Unclear |
| **Juan YANG**  **2011** |  | According to the method of random number table, they  were divided into a treatment group and a control  group | The random number and the associated  allocated treatment were kept in sequentially numbered  sealed opaque envelopes. | Placebo  needle provided participant-blinding effects with a  similar appearance to conventional needles | The assessors and statisticians  were unaware of the group allocation throughout the  study period | Did not fully report the data mentioned | Pre-specified  outcomes  reported | Not reported |
| **Risk of Bias** | HIGH | Low | Low | Low | Low | High | Low | Unclear |
| **Yi-jing JIANG**  **2011** |  | According to the method of random number table, they  were divided into a treatment group and a control  group | The random number and the associated  allocated treatment were kept in sequentially numbered  sealed opaque envelopes. | Placebo  needle provided participant-blinding effects with a  similar appearance to conventional needles | The assessors and statisticians  were unaware of the group allocation throughout the  study period | All analyses by  intension to  treat.  Participants lost  to follow-up  documented  with reason (ie death) if  possible. | Pre-specified  outcomes  reported | Not reported |
| **Risk of Bias** | UNCLEAR | Low | Low | Low | Low | Low | Low | Unclear |
| **You-hua ZENG**  **2015** |  | A total of 100 eligible participants were randomly  at a ratio of 1:1 by using random number table | Not reported | Participants and study personnel  were aware of the study-group  assignments | Not reported | Assumed  complete  outcome data  for all patients  as primary  outcome was  investigator  reported. | Pre-specified  outcomes  reported | Not reported |
| **Risk of Bias** | UNCLEAR | Low | Unclear | Unclear | Unclear | Low | Low | Unclear |
| **Zhi-cheng LIN**  **2014** |  | Using random number table randomly at a ratio of 1:1 | Not reported | Participants and study personnel  were aware of the study-group  assignments | Not reported | Amount of  missing  outcome data  was balanced  between the  treatment arms | Pre-specified  outcomes  reported | Not reported |
| **Risk of Bias** | UNCLEAR | Low | Unclear | Unclear | Unclear | Low | Low | Unclear |
| **Xue-mei WANG**  **2015** |  | A total of 80 eligible participants were randomly  at a ratio of 1:1 | Not reported | Participants and study personnel  were aware of the study-group  assignments | Not reported | Assumed  complete  outcome data  for all patients  as primary  outcome was  investigator  reported. | Pre-specified  outcomes  reported | Not reported |
| **Risk of Bias** | UNCLEAR | Unclear | Unclear | Unclear | Unclear | Low | Low | Unclear |
| **Rui-cong DING**  **2020** |  | The patients were  randomly assigned to a total of 44 patients. | Not reported | Participants and study personnel  were aware of the study-group  assignments | Not reported | Amount of  missing  outcome data  was balanced  between the  treatment arms | Pre-specified  outcomes  reported | Not reported |
| **Risk of Bias** | UNCLEAR | Unclear | Unclear | Unclear | Unclear | Low | Low | Unclear |
| **Qiang LIU**  **2016** |  | All patients were equally and randomly divided into  control and treatment groups. | Not reported | Participants and study personnel  were aware of the study-group  assignments | Not reported | Amount of  missing  outcome data  was balanced  between the  treatment arms | Pre-specified  outcomes  reported | Not reported |
| **Risk of Bias** | UNCLEAR | Unclear | Unclear | Unclear | Unclear | Low | Low | Unclear |
| **Bingxin WEI 2023** |  | According to the method of random number table, they  were divided into a treatment group and a control  group | Not reported | Placebo  needle provided participant-blinding effects with a  similar appearance to conventional needles | Not reported | Amount of  missing  outcome data  was balanced  between the  treatment arm | Pre-specified  outcomes  reported | Not reported |
| **Risk of Bias** | UNCLEAR | Low | Unclear | Low | Unclear | Low | Low | Unclear |
| **Xiyuan CHEN 2018** |  | method of random number table, they  were divided into a treatment group and a control  group | Not reported | Participants and study personnel  were aware of the study-group  assignments | Not reported | Amount of  missing  outcome data  was balanced  between the  treatment arm | Pre-specified  outcomes  reported | Not reported |
| **Risk of Bias** | UNCLEAR | Low | Unclear | Unclear | Low | Low | Low | Unclear |
| **Hongling YANG 2015** |  | method of random number table, they  were divided into a treatment group and a control  group | The random number and the associated  allocated treatment were kept in sequentially numbered  sealed opaque envelopes. | Participants and study personnel  were aware of the study-group  assignment | The assessors and statisticians  were unaware of the group allocation throughout the  study period | Amount of  missing  outcome data  was balanced  between the  treatment arm | Pre-specified  outcomes  reported | Not reported |
| **Risk of Bias** | UNCLEAR | Low | Low | Unclear | Low | Low | Low | Unclear |
| **Yang YANG 2019** |  | method of random number table, they  were divided into a treatment group and a control  group | Not reported | Participants and study personnel  were aware of the study-group  assignment | The assessors and statisticians  were unaware of the group allocation throughout the  study period | Amount of  missing  outcome data  was balanced  between the  treatment arm | Pre-specified  outcomes  reported | Not reported |
| **Risk of Bias** | UNCLEAR | Low | Unclear | Unclear | Low | Low | Low | Unclear |
| **Hongwei YUAN 2022** |  | method of random number table, they  were divided into a treatment group and a control  group | Not reported | Participants and study personnel  were aware of the study-group  assignment | The assessors and statisticians  were unaware of the group allocation throughout the  study period | Amount of  missing  outcome data  was balanced  between the  treatment arm | Pre-specified  outcomes  reported | Not reported |
| **Risk of Bias** | UNCLEAR | Low | Unclear | Unclear | Low | Low | Low | Unclear |

**Table 3 Results of Methodological quality evaluation**

| Author /Year | Item 1 | Item 2 | Item 3 | Item 4 | Item 5 | Item 6 | Item 7 | Recommendation |
| --- | --- | --- | --- | --- | --- | --- | --- | --- |
| Jing CHEN  2020 | + | ？ | ？ | + | + | + | ？ | ？ |
| Cai JIANG  2016 | + | ？ | ？ | ？ | + | + | ？ | ？ |
| Li-fang CHEN  2016 | + | + | ？ | + | + | + | ？ | ？ |
| Shu-hua WANG  2016 | + | + | ？ | + | + | + | ？ | ？ |
| Jian XIONG  2020 | + | ？ | + | + | + | + | ？ | ？ |
| Fan YANG  2017 | + | ？ | ？ | ？ | + | + | ？ | ？ |
| Rui YAO  2020 | + | ？ | ？ | ？ | + | + | ？ | ？ |
| Li HUANG  2021 | + | + | + | ？ | + | + | ？ | ？ |
| Li-tai SUN  2013 | + | ？ | ？ | ？ | - | + | ？ | - |
| Xiao-dong FENG  2013 | + | ？ | ？ | ？ | - | + | ？ | - |
| Lin LIU  2015 | + | + | ？ | ？ | + | + | ？ | ？ |
| Jia-jun KANG  2011 | + | + | + | + | + | + | ？ | ？ |
| Ya-nan ZHANG  2014 | + | ？ | ？ | ？ | + | + | ？ | ？ |
| Er-mei CAO  2015 | + | + | ？ | ？ | + | + | ？ | ？ |
| Juan YANG  2011 | + | + | + | + | - | + | ？ | - |
| Yi-jing JIANG  2011 | + | + | + | + | + | + | ？ | ？ |
| You-hua ZENG  2015 | + | ？ | ？ | ？ | + | + | ？ | ？ |
| Zhi-cheng LIN  2014 | + | ？ | ？ | ？ | + | + | ？ | ？ |
| Xue-mei WANG  2015 | ？ | ？ | ？ | ？ | + | + | ？ | ？ |
| Rui-cong DING  2020 | ？ | ？ | ？ | ？ | + | + | ？ | ？ |
| Qiang LIU  2016 | ？ | ？ | ？ | ？ | + | + | ？ | ？ |
| Bingxin WEI 2023 | + | ？ | + | ？ | + | + | ？ | ？ |
| Xiyuan CHEN 2018 | + | ？ | ？ | + | + | + | ？ | ？ |
| Hongling YANG 2015 | + | + | ？ | + | + | + | ？ | ？ |
| Yang YANG 2019 | + | ？ | ？ | + | + | + | ？ | ？ |
| Hongwei YUAN 2022 | + | ？ | ？ | + | + | + | ？ | ？ |

Note: Item 1: randomenzation;Item2 : allocation concealment; Item 3: blindness of participants and personnel； Item 4: blindness of outcome evaluator; Item 5: outcomes measured reliably; Item 6: selective reporting; Item 7: Other bias. +: Low Risk;?: Unclear Risk; -: High Risk

**Table 4 The effects of acupuncture on cognition and motor function**

| Outcome | N | Sample size | MD/SMD  (And 95%CI) | *I*^2^ value (%) | *P*-value | *P* _heterogeneity_ |
| --- | --- | --- | --- | --- | --- | --- |
| MoCA | 14 | 1157 | 1.78 [1.19, 2.37] | 62% | ＜0.00001 | 0.001 |
| MMSE | 16 | 1267 | 2.15 [1.21, 3.09] | 92% | ＜0.0001 | ＜0.00001 |
| P300 (PL) | 7 | 407 | 12.70 [5.64, 19.75] | 89% | 0.0004 | ＜0.00001 |
| P300 (Ams) | 7 | 407 | 1.37 [1.03, 1.70] | 0% | ＜0.00001 | 0.71 |
| LOTCA | 2 | 150 | 14.55 [11.87, 17.23] | 0% | ＜0.00001 | 0.47 |
| NIHSS | 2 | 301 | 1.12 [-0.82, 3.06] | 91% | 0.26 | 0.001 |
| FMA | 4 | 531 | 5.04 [-1.84, 11.93] | 78% | 0.15 | 0.004 |
| BI | 10 | 804 | 3.39 [0.54, 6.25] | 92% | 0.02 | ＜0.00001 |

**Note:** ①MoCA: Montreal Cognitive Assessment ②MMSE: Mini-Mental State Examination ③P300: potential 300 ④LOTCA: Loewenstein Occupational Therapy Cognitive Assessment ⑤NIHSS: National Institute of Health Stroke Scale ⑥FMA: Fuel-Meyer ⑦BI: Barthel Index

**Fig. 1. Meta-regression for year of publication, length of intervention and intervention method**


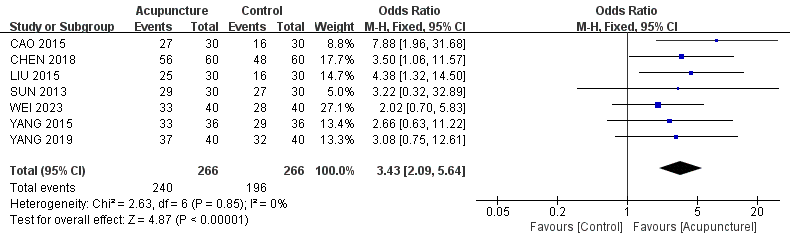


**Fig. 2. Forest plot of the effective rate.**

**Fig. 3. Egger and Begg’s test (MoCA)**

**Fig. 4. Egger and Begg’s test (MMSE)**


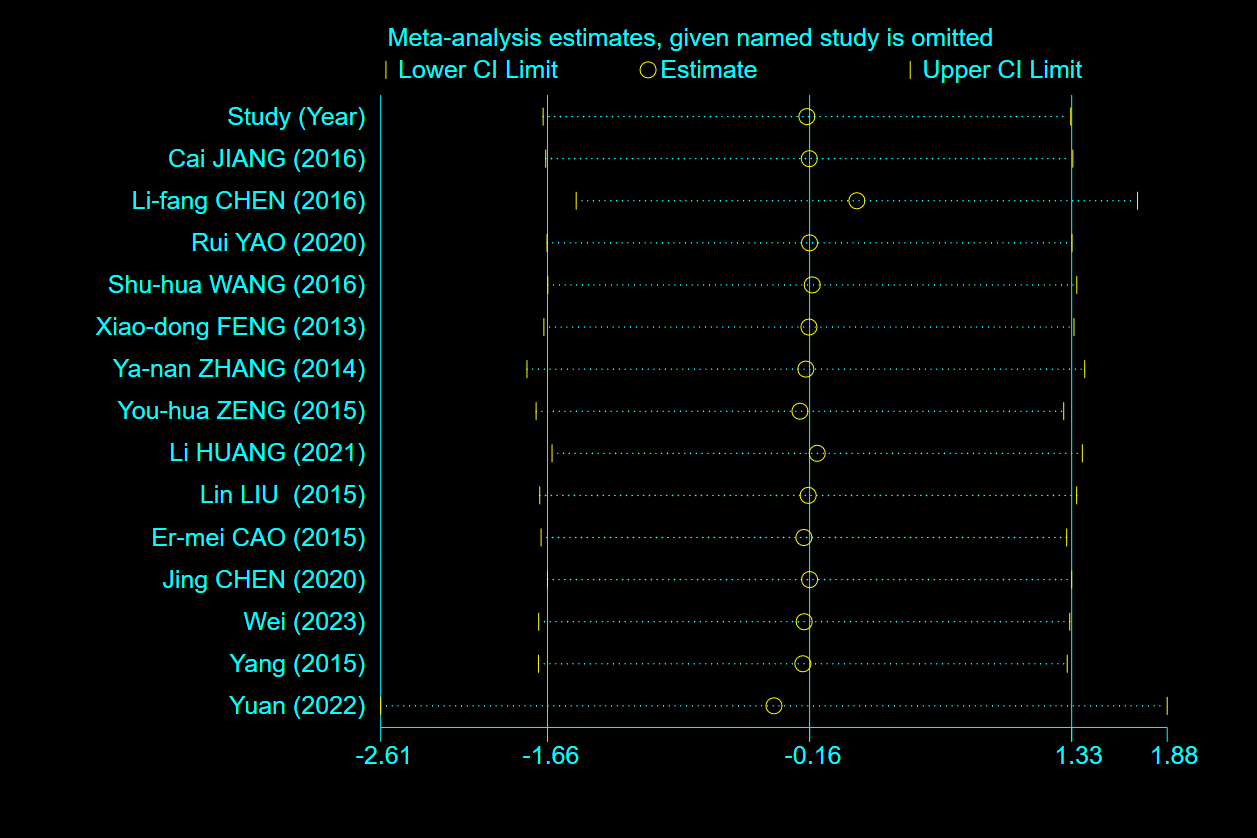


**Fig. 5. Sensitive analysis and robust examine: MoCA**


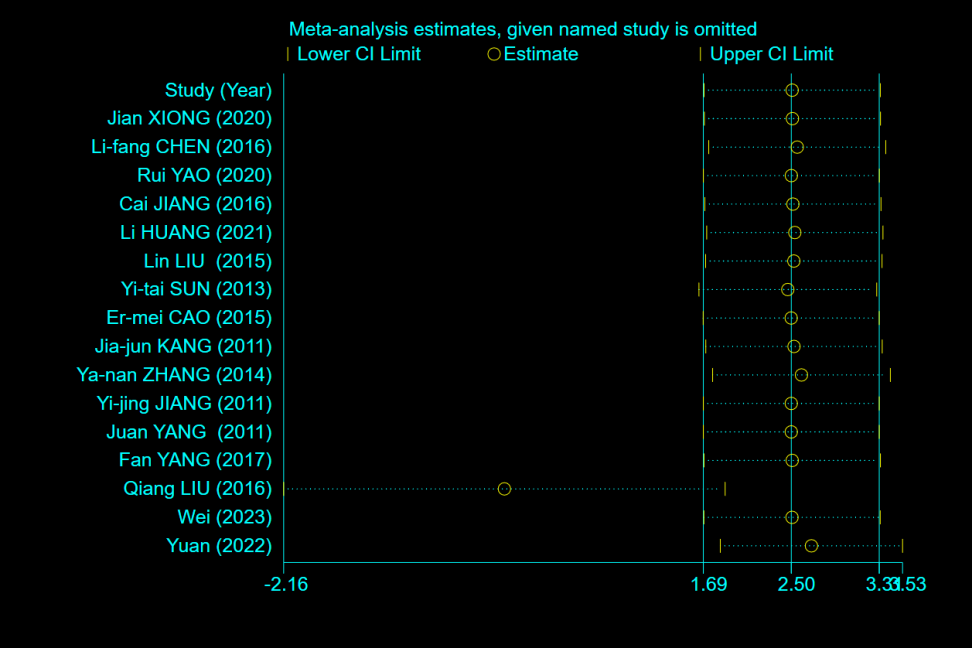


**Fig. 6. Sensitive analysis and robust examine: MMSE**
